# Cortex-specific transcriptome profiling reveals upregulation of interferon-regulated genes after deeper cerebral hypoperfusion in mice

**DOI:** 10.3389/fphys.2023.1056354

**Published:** 2023-03-13

**Authors:** Zengyu Zhang, Zimin Guo, Zhilan Tu, Hualan Yang, Chao Li, Mengting Hu, Yuan Zhang, Pengpeng Jin, Shuangxing Hou

**Affiliations:** ^1^ Department of Neurology, Shanghai Pudong Hospital, Fudan University, Shanghai, China; ^2^ School of Pharmacy, Hubei University of Science and Technology, Hubei, China; ^3^ Department of Vascular Surgery, Shanghai Pudong Hospital, Fudan University, Shanghai, China; ^4^ Department of Chronic Disease Management, Shanghai Pudong Hospital, Fudan University, Shanghai, China

**Keywords:** bilateral carotid artery stenosis, cerebral cortex, RNA-seq, type I interferon, chronic cerebral hypoperfusion (CCH)

## Abstract

**Background:** Chronic cerebral hypoperfusion (CCH) is commonly accompanied by brain injury and glial activation. In addition to white matter lesions, the intensity of CCH greatly affects the degree of gray matter damage. However, little is understood about the underlying molecular mechanisms related to cortical lesions and glial activation following hypoperfusion. Efforts to investigate the relationship between neuropathological alternations and gene expression changes support a role for identifying novel molecular pathways by transcriptomic mechanisms.

**Methods:** Chronic cerebral ischemic injury model was induced by the bilateral carotid artery stenosis (BCAS) using 0.16/0.18 mm microcoils. Cerebral blood flow (CBF) was evaluated using laser speckle contrast imaging (LSCI) system. Spatial learning and memory were assessed by Morris water maze test. Histological changes were evaluated by Hematoxylin staining. Microglial activation and neuronal loss were further examined by immunofluorescence staining. Cortex-specific gene expression profiling analysis was performed in sham and BCAS mice, and then validated by quantitative RT-PCR and immunohistochemistry (IHC).

**Results:** In our study, compared with the sham group, the right hemisphere CBF of BCAS mice decreased to 69% and the cognitive function became impaired at 4 weeks postoperation. Besides, the BCAS mice displayed profound gray matter damage, including atrophy and thinning of the cortex, accompanied by neuronal loss and increased activated microglia. Gene set enrichment analysis (GSEA) revealed that hypoperfusion-induced upregulated genes were significantly enriched in the pathways of interferon (IFN)-regulated signaling along with neuroinflammation signaling. Ingenuity pathway analysis (IPA) predicted the importance of type I IFN signaling in regulating the CCH gene network. The obtained RNA-seq data were validated by qRT-PCR in cerebral cortex, showing consistency with the RNA-seq results. Also, IHC staining revealed elevated expression of IFN-inducible protein in cerebral cortex following BCAS-hypoperfusion.

**Conclusion:** Overall, the activation of IFN-mediated signaling enhanced our understanding of the neuroimmune responses induced by CCH. The upregulation of IFN-regulated genes (IRGs) might exert a critical impact on the progression of cerebral hypoperfusion. Our improved understanding of cortex-specific transcriptional profiles will be helpful to explore potential targets for CCH.

## Introduction

Chronic cerebral hypoperfusion (CCH), refers to a group of brain dysfunction syndromes caused by long-term decreased cerebral perfusion (Ciacciarelli et al., 2020). CCH can cause damage to the cerebral microcirculation, and then aggravate each other, eventually resulting in a wide variety of cerebral disorders such as lacunar infarction, subcortical microinfarction and cortical lesions (Iadecola, 2013). CCH-induced neuroinflammation is proved to be one of the most significant pathophysiological mechanisms (Hase et al., 2017; Suzuki et al., 2021). Proliferating and activated microglia produce cytotoxic factors and inflammatory mediators (Miyanohara et al., 2018; Kakae et al., 2019; Du et al., 2020; Liu et al., 2020), while reactive astrogliosis promotes the expression of inflammatory mediators and participates in neuroinflammatory processes, thus forming a vicious circle following hypoperfusion (Magami et al., 2019). The bilateral carotid artery stenosis (BCAS) mouse model has been evaluated as one of the most germane rodent models of cerebral hypoperfusion (Gao et al., 2019). Neuropathological changes of CCH presenting with white matter lesions (bilateral 0.18 mm BCAS, including demyelination and inflammation), and gray matter damage due to deeper cerebral hypoperfusion (BCAS using 0.16/0.18 mm microcoils) (Miki et al., 2009; Washida et al., 2019). And cortical lesions are considered to be the key pathological changes associated with deeper cerebral hypoperfusion (Miki et al., 2009).

Of note, the underlying cellular and molecular mechanisms of cortex damage and glial activation are largely unknown after hypoperfusion. Research on significant genes and molecular pathways involved in CCH will help uncover potential mechanisms and provide new therapeutic targets. RNA sequencing (RNA-seq), a promising strategy to identify specific changes with unbiased profiling in the transcriptome, has the ability to detect large-scale gene expressions, to measure a greater number of gene transcripts, and to examine differences in sequence (Liu et al., 2021).

Here, we successfully established the 0.16/0.18 mm BCAS-hypoperfusion model with cortical lesions based on prior studies (Miki et al., 2009). Microglia manifested a state of neuroinflammatory activation in the cerebral cortex in BCAS mice. RNA-seq was used to further elucidate the cortex-specific gene-expression profiles after hypoperfusion, followed by qRT-PCR and immunohistochemistry (IHC) validation. We aimed to investigate the cortex-specific transcriptional changes in order to improve our understanding of the molecular mechanisms of the neuroimmune response to chronic cerebral ischemia injury.

## Materials and methods

### Animal experimental design

The animal experiments in this study were in compliance with the ARRIVE guidelines (Kilkenny et al., 2011). Adult male C57BL/6 J mice (10–11 weeks, weight 23 g–27 g) were provided by Beijing Vital River Laboratory Animal Technology. Mice were given access to food and water *ad libitum*, and housed under SPF conditions in IVC cages. After one-week acclimation, the mice were divided into 2 groups by random number method: the BCAS group and the sham group. The experimental unit was single mouse. A total of 92 mice were used in this study, 15 mice failed to survive the BCAS operation. The remaining 77 mice were used for the entire experiment. Total 18 mice were used in the Morris water maze test and 4-week pathological staining (group sham, *n* = 10; group BCAS, *n* = 8), 22 mice in the cerebral blood flow measurement (group sham, *n* = 10; group BCAS, *n* = 12), 15 mice in the 3-week pathological staining (group sham, *n* = 7; group BCAS, *n* = 8), 8 mice in the RNA-seq (group sham, *n* = 4; group BCAS, *n* = 4), and 14 mice in the qRT-PCR experiment (group sham, *n* = 7; group BCAS, *n* = 7).

### Bilateral carotid artery stenosis procedure

BCAS surgery was performed as described with minor modification (Shibata et al., 2004; Miki et al., 2009). Briefly, the mice were anesthetized with 2% isoflurane delivered in medical oxygen by a facemask. Then both common carotid arteries (CCAs) were exposed and isolated from the vagus nerves. Microcoils, inner diameter of 0.16 mm and 0.18 mm (Anruike Biotechnology, Xi’an, China), were applied to the surgical procedure. The 0.16 mm microcoil was wrapped around the right CCA, as described elsewhere (Zhou et al., 2022). After one hour, the 0.18 mm microcoil was twined around the left CCA. Sham-treated animals were exposed to identical procedures with the exception that microcoils were not placed around the arteries.

### Laser speckle contrast imaging

Real-time two-dimensional cerebral blood flow (CBF) was measured using laser speckle flowmetry (LSF, RFLSI III, RWD, China) in sham and BCAS mice 4 weeks postoperation as previously reported (Li C et al., 2022). Briefly, the mice were anesthetized and placed in a prone position. The skull was exposed by an incision of the skin along the midline of the scalp. Then, the LSF was elevated to an appropriate height above the skull surface. And a whole-brain scan was performed using the LSF. To assess CBF changes, regions of interest (ROIs) were manually selected and the data were analyzed using LSCI_V 1.0.0 software (RWD, China). Body temperature was maintained throughout the experiment.

### Morris Water Maze test

Cognitive function was determined using the water maze test as previously described with some modifications (Ma et al., 2013). Mice were first subjected to the positioning and navigation test for five consecutive days and four trials per day after BCAS surgery. The swimming speed and escape latency (water maze-learning) were recorded. The probe test (water maze-memory) was conducted in the absence of the platform with a cut-off time of 120 s on the sixth day. The first latency to the platform, time spent in quadrant, frequency of crossing the platform area and track plots were collected. Data were analyzed using the Ethovision XT 15 software (Noldus Inc.).

## Tissue preparation

At 3 or 4 weeks post-surgery, mice were anesthetized with 2% isoflurane and decapitated. Brains were removed, post-fixed at 4°C overnight in 4% formaldehyde in PBS and dehydrated in 30% glucose at 4°C till sink. Coronal slices at 50 µm thickness were prepared using a freezing microtome (CM 1900; Leica, Germany).

### Hematoxylin staining

We performed hematoxylin staining using the Solarbio kit (G1120) with minor modification. Because these changes of phenotypes could be captured without cytoplasm staining by eosin (Cao et al., 2018; Liu et al., 2018). For staining process, brain slices were fixed in methanol and stained with hematoxylin. Excess background stain was removed with a quick rinse in 1% HCl in alcohol, followed by treatment with the differentiation buffer. The slides were passed through 70% alcohol and anhydrous alcohol, and mounted with xylene mountant. Images were taken by digital slide scanner (Pannoramic DESK, P-MIDI, 3D HISTECH, Hungary). A whole-brain scan was performed for five brain slices from 7-8 animals in each group. Field of view of the left or right hemisphere was selected. Infarct area was characterized by monocyte or microglial nucleus accumulation, and measured using the CaseViewer 2.4 software. “Draw a closed polygon” of “Annotations” in the CaseViewer software was used to manually define the infarct area. And the software automatically calculated the area values inside the selection. Cerebral infarction area (%) was defined as (infarct area)/(whole-brain area) x100%.

### Immunofluorescence staining

For immunofluorescence staining, free-floating sections were washed in 1 × PBS and incubated with a blocking buffer [1% Triton X-100 in PBS containing 5% normal goat serum (Thermo, 16210064)] for 1 h. Sections were treated with primary antibodies against Iba-1 (Abcam, ab178846), NeuN (Sigma, MAB377A5), Iba-1 (Abcam, ab5076) and CD68 (Abcam, ab283654) in blocking solution at 4°C overnight. These brain sections were then incubated with secondary antibodies [(Goat anti rabbit Alexa Fluor 647, Jackson, 111–605–003), (Donkey anti rabbit Alexa Fluor 488, Invitrogen, A-21206), (Donkey anti goat Alexa Fluor 594, Jackson, 705–585–003)] for 1 h, followed by DAPI (Thermo, 62,248, 1:10,000 dilution) counterstaining for 15 min. NC means negative control without the primary antibody. Background staining is that of the secondary antibody alone. Sections were scanned with an Olympus slide scanner (VS120-L100). High resolution images were captured from the confocal microscopy (AIR-MP, Nikon). Images were processed using the ImageJ software. Five brain slices were chosen for each animal, and a whole-brain scan was observed in each section. Field of view of the left or right hemisphere was selected. Activate microglia was characterized by stronger fluorescence intensity when compared to rest microglia (Lim et al., 2018). As previously reported, for the assessment of activated microglia cells, areas with positive signal were manually outlined (“Measures a freehand polygon” of “Freehand Polygon” in the OlyVIA software), and a measure of the area of coverage by positive signal (percent of the total area) was noted for each region (Ivanova et al., 2020). The area values were automatically calculated by the software. The relative activation area (%) was defined as (activation area)/(whole-brain area) x100%.

### Immunohistochemistry staining

Immunohistochemistry (IHC) was performed with frozen sections. The slides were stained with IFITM3 (11714-1-AP, Proteintech) primary antibody, followed by incubation with HRP-conjugated anti-rabbit antibody (RC0080R, Record Biology). Then sections were developed using DAB Peroxidase Substrate. Stained slides were counter-stained with hematoxylin and cover-slipped for review. In the negative control (NC) group, the primary antibody was not added and the rest of steps were kept the same. Image acquisition was performed on the Olympus Slideview VS200 at 20-fold magnification. The color of DAB staining was brown, while the color of hematoxylin was violet and the colors could be distinguished in the microscope. The taken images were processed with the OlyVIA (Olympus) software.

### RNA extraction, quantification and qualification

Total RNA of each sample was extracted from the right cerebral cortex according to the instruction manual of the TRIzol Reagent (Life technologies, California, United States). RNA concentration and purity was measured using NanoDrop 2000 (Thermo Fisher Scientific, Wilmington, DE).

### RNA-seq library construction

For RNA sample preparations, a total amount of 1 μg RNA per sample was used as the input material (*n* = 1 mouse/sample). Sequencing libraries were generated using NEBNext UltraTM RNA Library Prep Kit for Illumina (NEB, United States) following manufacturer’s recommendations. Briefly, mRNA was purified from total RNA, followed by fragmentation, first-strand and second-strand synthesis. cDNA then went through end-repair, A-addition, strand-specific ligation, and PCR amplification. At last, PCR products were purified (AMPure XP system) and library quality was assessed on the Agilent Bioanalyzer 2,100 system.

### RNA sequencing and data analysis

150 bp nucleotides were used for the paired-end sequencing on an Illumina NovaSeq 6,000. Raw data were first evaluated by FastQC for quality control (Sequencing depth and the quality control summary of RNA-seq data are presented in Supplementary Table S1, Supplementary Material S11). Adapter sequences, low-quality reads, and too short reads were removed with Trim-galore to obtain clean data. Next, sequencing reads were aligned to annotated RefSeq genes in the mouse reference genome (UCSC mm 10) with HISAT2 (Stephens et al., 2021), and Samtools v1.9 was used to sort and build the alignment file index. Then, gene counts were generated using the FeatureCounts v1.6.3. And raw transcript counts were normalized, log_2_ transformed, and analyzed using limma to identify genes that were differentially regulated. Gene set enrichment analysis (GSEA) for “Biological Process (BP)” was performed with the GSEA software (version 3.0) from the Broad Institute. We used FDR < 0.05 and |Log2FC| > 1 to define differentially expressed genes (DEGs) between sham and BCAS mice. We further performed ingenuity pathway analysis (IPA) to predict potential upstream regulators and canonical pathways of DEGs.

### Quantitative real-time PCR (qRT-PCR)

Total RNA was first reverse transcribed using Evo M-MLV RT Kit (AG11711, Accurate Biology), and qRT-PCR (Thermofisher, Applied Biosystems QuantStudio5) was then performed using SYBR^®^ Green Premix qPCR Kit (AG11718, Accurate Biology). The primer sequences were listed in Supplementary Material S12. Data were normalized to GAPDH as a reference gene. Relative expression values were calculated using the 2^−ΔΔCT^ method.

### Statistical analysis

All experiments and data analysis were conducted under investigator-blinded conditions. Statistical analyses were performed using Prism v8 for Windows (GraphPad Software). All data were expressed as mean ± SEM. Data were analyzed using paired *t*-test for comparisons of 0.18 mm side and 0.16 mm side in BCAS group samples (two-tailed). When comparisons were made between sham and BCAS groups, an unpaired *t*-test was performed (two-tailed). Significance level was set as *p* < 0.05. *p < 0.05, **p < 0.01, ***p < 0.001.

## Results

### Cerebral blood flow and behavioral alterations after deeper cerebral hypoperfusion

**FIGURE 1:**
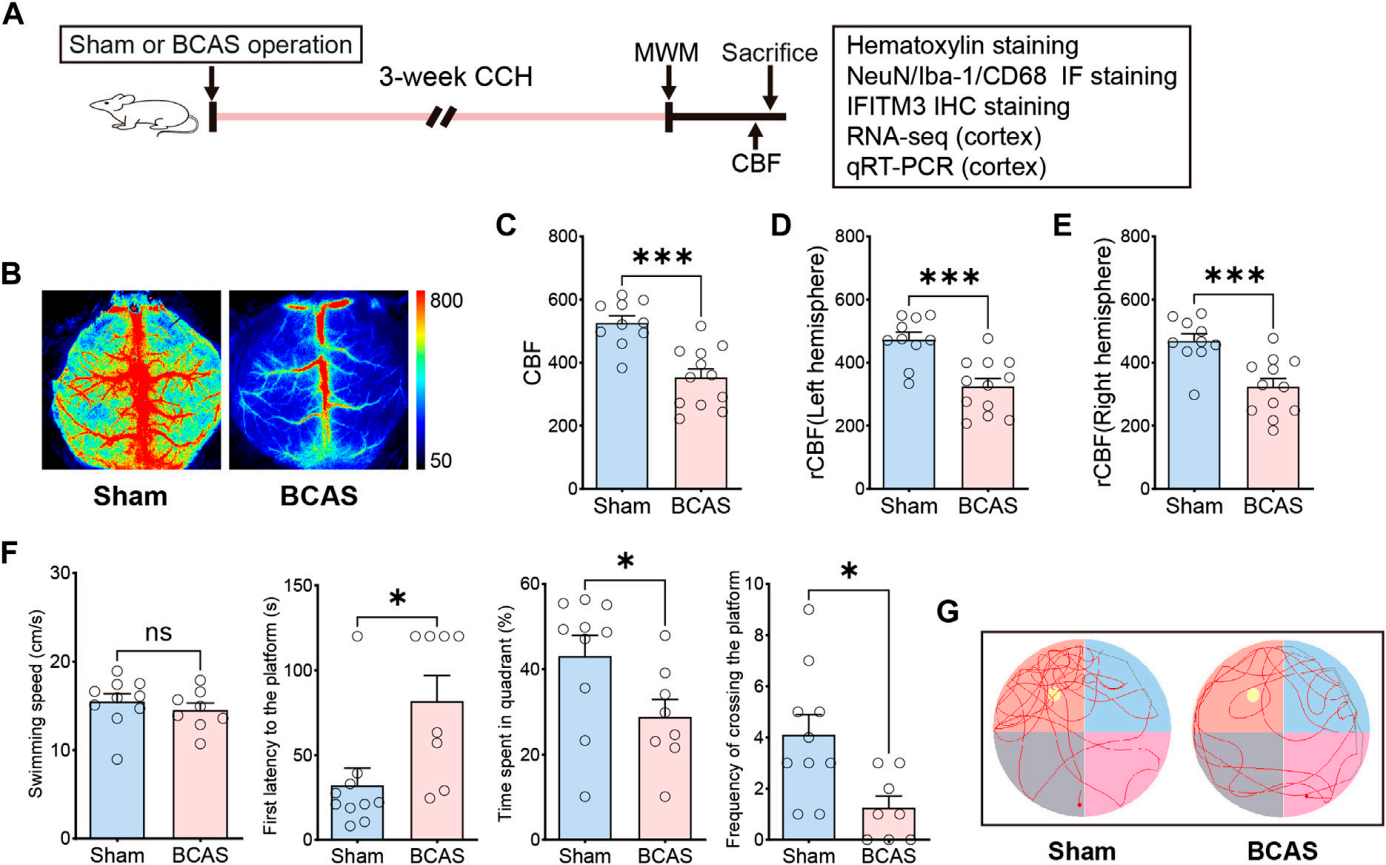
Changes of CBF and behavioral phenotype after severe cerebral hypoperfusion. **(A)** Flow chart of the experiment. BCAS, bilateral carotid artery stenosis. CCH, chronic cerebral hypoperfusion. MWM, Morris water maze test. Rcbf, regional cerebral blood flow. IF, Immunofluorescence staining. IHC, Immunohistochemistry staining. **(B)** Representative laser speckle contrast image on sham and BCAS group at 4 weeks postoperation. Bar plots for whole brain **(C)** left hemi-sphere **(D)**, right hemisphere **(E)** of CBF in sham and BCAS mice (n = 10–12 mice per group). **(F)** Average swimming speed, first latency to the platform, time spent in quadrant (%) and frequency of crossing the platform area of sham and BCAS mice in MWM probe test (n = 8–10 mice per group). **(G)** Representative track plots of sham and BCAS mice in MWM probe test. Data are expressed as mean ± SEM. Unpaired t test (two-tailed), *p < 0.05, **p < 0.01. ***p < 0.001 versus sham.

CBF changes could be monitored by using LSF. Consistent with the previous studies, we found that there was a significant reduction of CBF on the left and right side of brain at the fourth week after BCAS (Figures 1B–E). And the 0.16 mm stenosis led to an approximate 31% decrease in average CBF compared to the right hemisphere of sham mice. In order to evaluate changes in spatial cognitive function after prolonged hypoperfusion in the current BCAS model, we first conducted Morris Water Maze test in the two groups. Results suggested that the BCAS model did not induce motor dysfunction as evidenced by no difference in swimming speed between these two groups of mice either in learning phase or in memory phase (Supplementary Material S1; Figure 1F). In addition, BCAS mice showed impaired spatial learning ability compared with sham mice postoperation (Supplementary Material S1). Furthermore, compared with sham group, the BCAS group spent significantly less time in the target quadrant, accompanied by a reduction in the number of crossing the platform in the probe trial (Figures 1F, G), indicating spatial cognitive deficits post deeper cerebral hypoperfusion.

### Histological changes in different sections following deeper cerebral hypoperfusion

**FIGURE 2:**
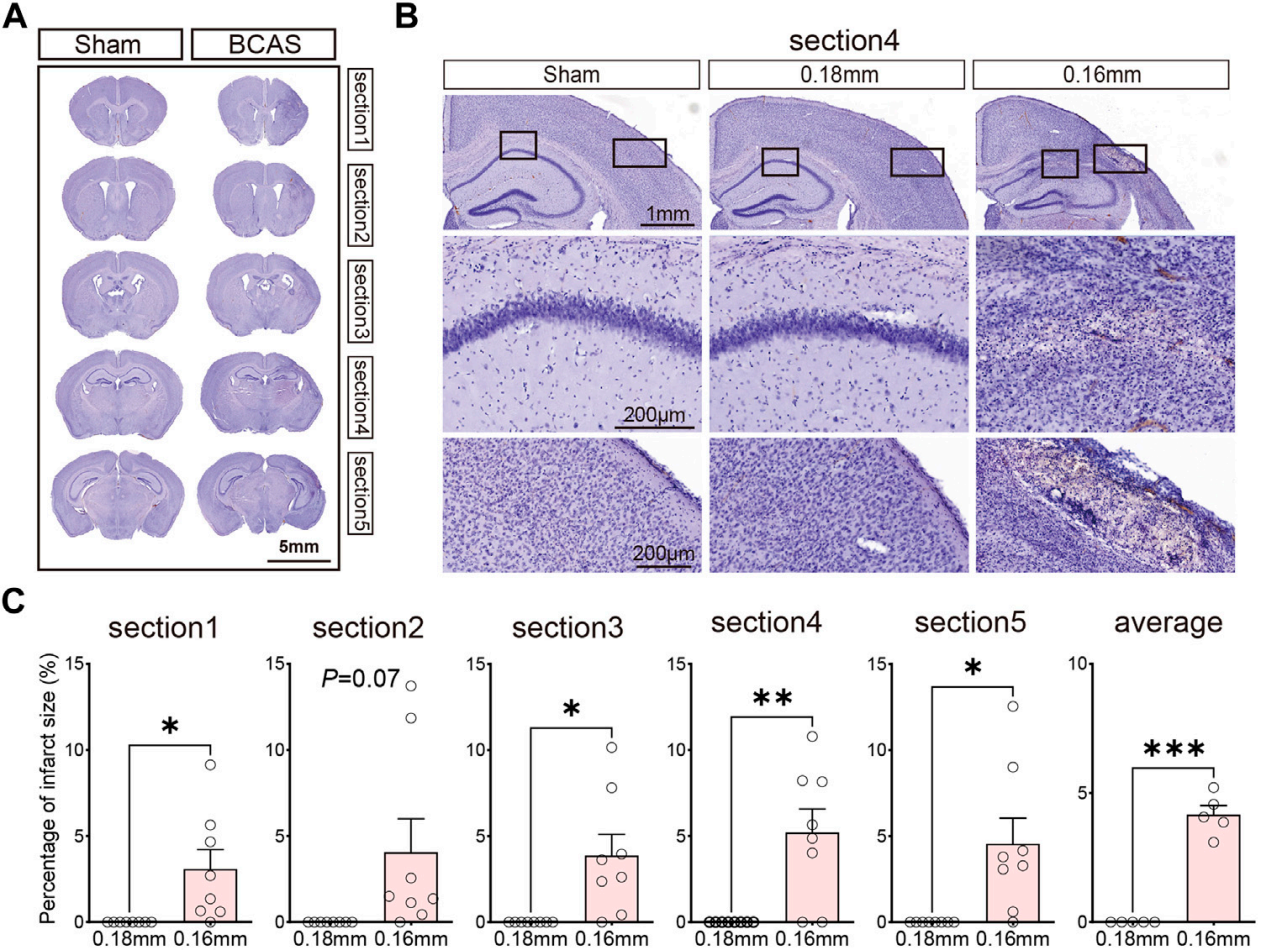
**(A)** Hematoxylin-stained consecutive coronal slices to detect brain injury after 3 weeks cerebral hypoperfusion. **(B)** Representative images depicting the morphology changes of cortex and hippocampus. **(C)** Bar plots for percentage of infarct area of each section in BCAS mice. Average, each point represents the mean value of per section of 8 mice. Data are expressed as mean ± SEM. Paired t test (two-tailed), *p < 0.05, **p < 0.01. ***p < 0.001 versus 0.18 mm side, n = 7–8 mice for each group.

Previous research has shown that intensity of CCH determines white/gray matter injury in mice. We evaluated the histological changes in different sections by Hematoxylin staining after BCAS- hypoperfusion (Figure 2; Supplementary Materials S2, S3). On the side of 0.16 mm stenosis, the entire hemisphere was atrophied, and the cortex was thinned (Figure 2A). Significant neuronal loss was seen in the cortex and CA1 region of the hippocampus (Figure 2B; Supplementary Material S2) on the 0.16 mm side. In addition, we analyzed the percent infarct size of the 0.18 mm side and 0.16 mm side of the BCAS group for five sections. The mean percent infarct size in each section was also compared. Results showed the infarct was obvious on the 0.16 mm side, the difference was statistically significant (Figure 2C). And the 0.16 mm microcoil resulted in an approximate 10.5% increase in average percent of infarct area per section compared to the 0.18 mm stenosis side. Thus, the BCAS-hypoperfusion induced obvious histological injury on the 0.16 mm stenosis side. Our results are consistent with previous reports, and for the first time, we have performed consecutive coronal brain sections to provide a comprehensive basis for studying neuropathological changes in deeper cerebral hypoperfusion.

### Cortical lesions involving microglial activation and neuronal loss following deeper cerebral hypoperfusion

**FIGURE 3:**
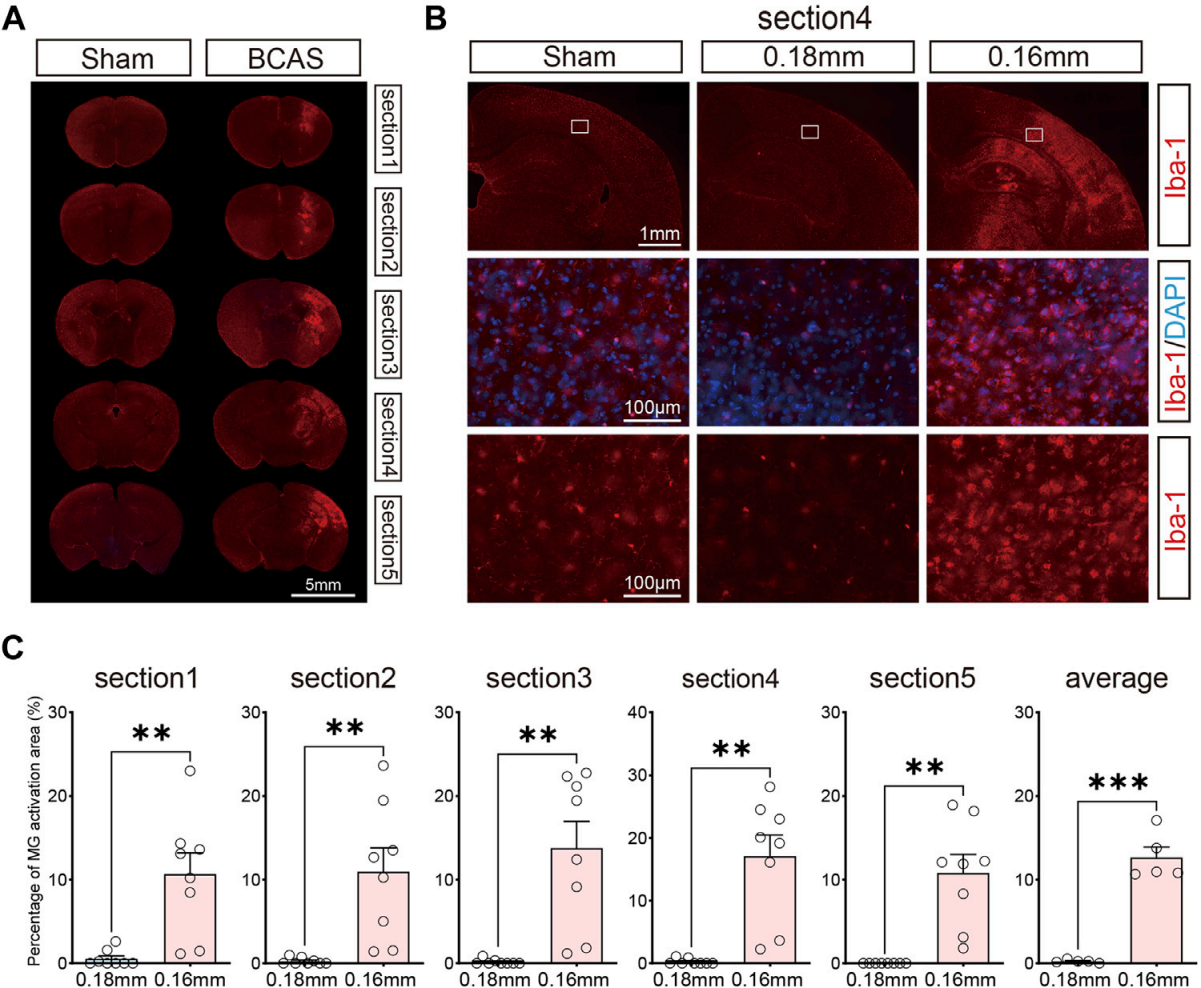
Activation of microglia due to deeper cerebral hypoperfusion. **(A)** Consecutive coronal brain sections labeled with Iba-1 3 weeks post BCAS operation. **(B)** Representative pictures of Iba-1 labeled sections revealing obvious microglial activation of cerebral cortex. **(C)** Bar plots for percent of microglial activatin area of each section in BCAS mice. Average, each point represents the mean value of per section of 8 mice. MG, microglia. Paired t test (two-tailed), **p < 0.01. ***p < 0.001 versus 0.18 mm side, n = 7–8 mice for each group.

We next examined microglial activation after BCAS-hypoperfusion, as microglial activation is a key element in initiating and perpetuating inflammatory responses to ischemia. We detected the Iba-1 (the microglia marker) labeled sections by IF staining in the 0.16/0.18 mm BCAS model. General presentation of microglial activation can be seen in Figure 3A. Hyperactivation of microglia occurred mostly in areas of neuronal injury, including the striatum, cerebral cortex and DG region of the hippocampus on the 0.16 mm side (Figure 3B; Supplementary Material S4). Also, we analyzed the percentage of microglial activation area of the 0.18 mm side and 0.16 mm side of the BCAS group for five sections. The mean percent activation area of each section was also compared. Results showed that microglial activation was obvious on the 0.16 mm side, and the difference was statistically significant (Figure 3C). Then, CD68/Iba1 double-staining was performed to further investigate the activated microglia after BCAS-hypoperfusion. We found that part of microglia/macrophage showed high expression on the 0.16 mm stenosis side of BCAS mice, instead of sham group or 0.18 mm side (Supplementary Material S5). The results suggested that some of activated microglia/macrophage displayed phagocytosis phenotypes after BCAS-hypoperfusion, which was consistent with the previous study.

**FIGURE 4:**
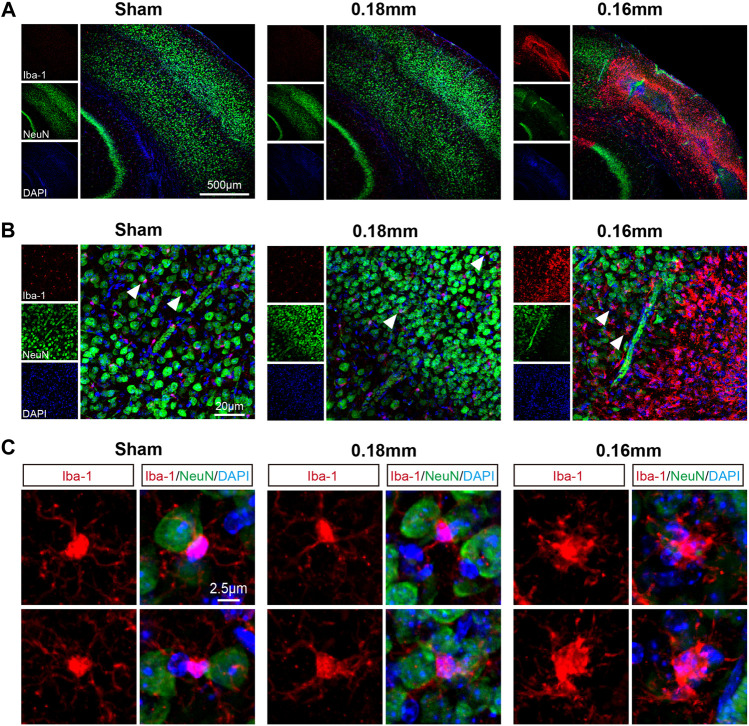
Microglial activation is accompanied by neuronal loss in the cerebral cortex following chronic hypoperfusion. **(A)** Representative confocal images labeles with Iba-1/NeuN. **(B–C)** Higher resolution of representative images showed that the activated microglia lost most normal morphology and became amoeboid-like phenotype with the typical characteristics of phagocytosis around the areas of neuron loss.

Cortical lesions presented random distribution after BCAS-hypoperfusion. We then took the cortex as region of interest and used laser confocal scanning for further investigation. Colocalization of Iba-1 and NeuN (the neuron marker) revealed that the activated microglia lost most of the normal morphology and became amoeboid-like phenotype with the typical characteristics of phagocytosis around the areas of neuronal loss (Figures 4A–C).

### Cortex-specific transcriptional analysis for up- and downregulation pathways after deeper cerebral hypoperfusion

**FIGURE 5:**
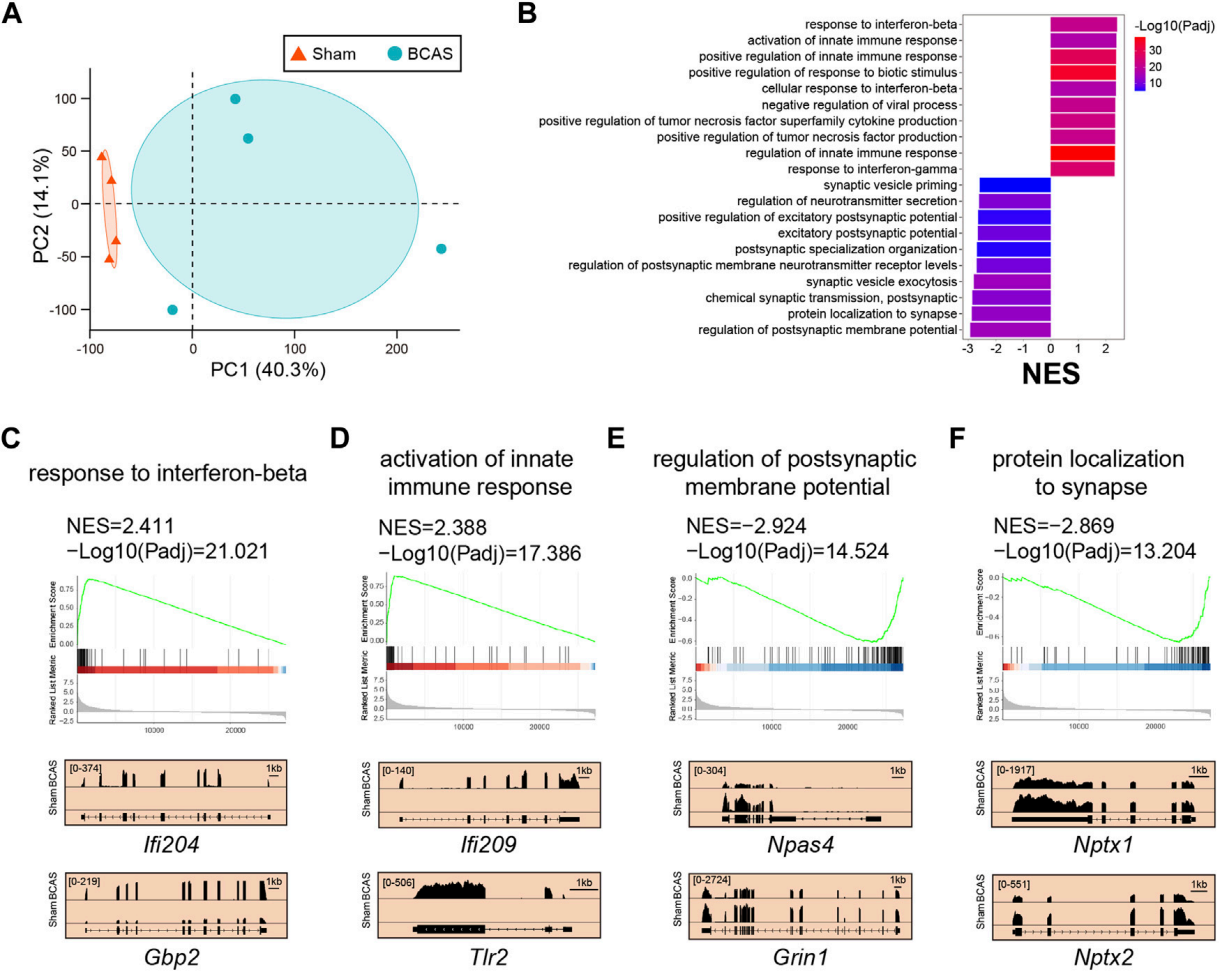
Cortex-specific pathway analysis of trascriptome changes by limma after BCAS-hypoperfusion. **(A)** PCA plot of cortex-specific RNA-seq obtained from sham and BCAS mice. N = 4 for each group. **(B)** Up-regulated and down-regulated functional pathways analyzed by GSEA (BCAS vs. Sham) NES, normalized enrichment score. Notice high enrichment for IFN-beta signaling pathways.. **(C–F)** (Up) Enrichment plots and (Bottom) IGV map tracks for representative genes in the up-regulated and down-regulated pathways. Notice increases of RNA-seq signal for IFN-regulated genes (*IFi204*, *Gbp2*) and activation of innate immune genes (*Ifi209*, *Tlr2*) in BCAS group compared to sham mice, accompanied by decreases of RNA-seq signal for neuronal-activity related genes (*Npas4*, *Grin1*, *Nptx1*, *Nptx2*).

Here, we focused on cortex-specific transcriptional changes on the 0.16 mm stenosis side post BCAS-hypoperfusion. We analyzed cortex-specific RNA-seq data for 4 biological replicates by limma at 3 weeks post-surgery. Principal component analysis (PCA) revealed a clear separation between the BCAS and sham mice (Figure 5A), indicating a unique transcriptome signature of cortex after hypoperfusion. The GSEA does not set a difference threshold and is able to detect weak but consistent trends. GSEA analysis showed multiple extremely important upregulated pathways (BCAS vs. Sham) associated with interferon (IFN)-mediated biological processes. The top four were “response to interferon-beta (*Ifi204*, *Gbp2*) (Padj: 9.53E-22; NES: 2.41)”, “activation of innate immune response (*Ifi209*, *Tlr2*) (Padj: 4.12E-18; NES: 2.39)”, “positive regulation of innate immune response (*Itgp*, *Il12b*) (Padj: 8.39E-30; NES: 2.37)”, and “positive regulation of response to biotic stimulus (*Hmgb2*, *Fadd*) (Padj: 1.11E-35; NES: 2.37)” (Figures 5B–D; Supplementary Material S6). In addition, consistent with neuronal loss in pathology, GSEA also showed several significantly important downregulated pathways (BCAS vs. Sham) related to neuronal-activity. The top four were “regulation of postsynaptic membrane potential (*Npas4*, *Grin1*) (Padj: 3.00E-15; NES: −2.92)”, “protein localization to synapse (*Nptx1*, *Nptx2*) (Padj: 6.26E-14; NES: −2.87)”, “chemical synaptic transmission, postsynaptic (*Rgs4*, *Stx1a*) (Padj: 1.42E-12; NES: −2.85)”, and “synaptic vesicle exocytosis (*Nrn1*, *Chrnb3*) (Padj: 5.06E-15; NES: −2.80)” (Figures 5E, F; Supplementary Material S6). Also, the data analyzed by the “DESeq2 package” were shown in Supplementary Materials S7–S9. Notably, similar results were obtained by the two methods. Together, our data suggested that transcriptional activation of IFN-regulated genes (IRGs) and transcriptional repression of genes in neuronal-activity pathway after deeper chronic hypoperfusion.

### Ingenuity pathway analysis of DEGs predicted potential upstream regulators and canonical pathways post deeper cerebral hypoperfusion

**FIGURE 6:**
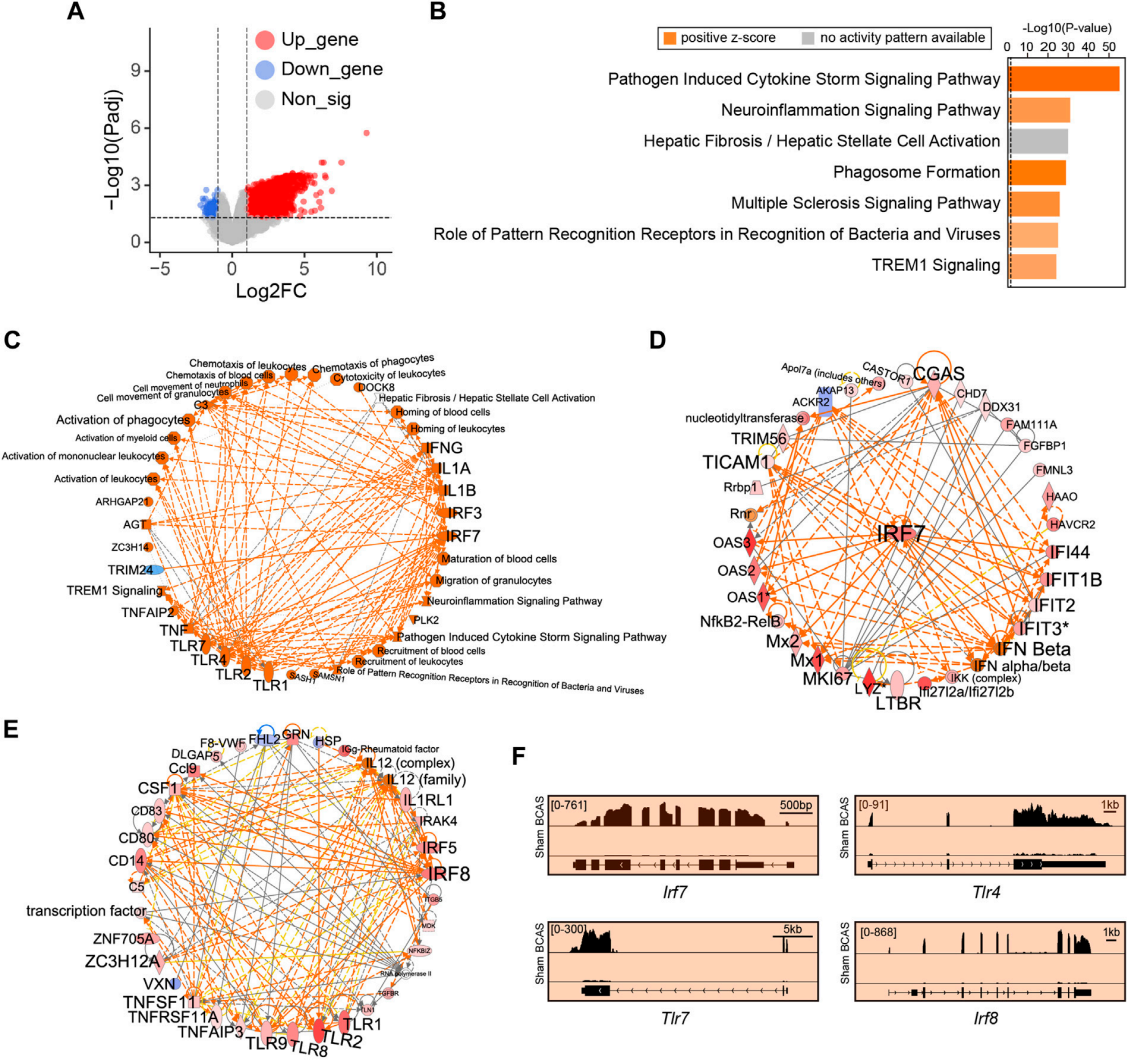
Cortex-specific IPA analysis of DEGs by limma post cerebral hypoperfusion. **(A)** Volcano plots indicated genes with differential expression as defined by an adjusted P value < 0.05 and $a\;\left|\mathrm{Log}2FC\right|$ > 1 in the cerebral cortex after operation (BCAS vs. Sham). Points highlighted in red: significant differentially expressed gene: Points highlighted in blue: significant differentially down-regulated expressed gene; Gray dot: non-significant differentially expressed gene. **(B)** Bar plot showing the top canonical pathways significantly enriched in the BCAS-hypoperfusion mice. Orange bars; positive regulatory pathway; Gray bar: no activity pattern available. **(C–E)** Upstream regulatory analysis of DEGs revealing IRF7-related hub genes are pointed to be the top upstream regulators in IPA effect networks. TLRs also participated in and coordinately regulated genes of IFN signaling. **(F)** IGV map tracks for representative genes in the too canonical pathways. Notice increases of RNA-seq signal for IFN-regulated genes (*Irf7*, *Irf8*, *Tlr4*, and *Tlr7*) in BCAS mice compared to sham group.

Here, gene sets with strong alterations were analyzed, followed by a high cutoff setting. Compared to sham group, we identified 2,145 DEGs (FDR < 0.05, |Log2FC| > 1) in the cortex following BCAS operation. Of these 2,145 DEGs, 1894 (88.3%) genes were upregulated and 251 (11.7%) genes were downregulated (Figure 6A; Supplementary Table S2). IPA analysis was conducted to predict potential upstream regulators and canonical pathways. We found that immune-related biological processes including “pathogen induced cytokine storm signaling pathway”, “neuroinflammation signaling pathway”, “phagosome formation”, and “multiple sclerosis signaling pathway” were significantly enriched and upregulated, and were mostly positively regulated (Figure 6B; Supplementary Table S3). Notably, the hepatic fibrosis/hepatic stellate cell activation related pathway was also significantly enriched after hypoperfusion, but its effect manifested as no activity pattern available. In addition, we found that the Toll-like receptors (TLRs)-mediated IFN signaling was predicted to exert critical regulatory effect after BCAS-hypoperfusion (Figure 6C). Specifically, TLR pathways related genes including *Tlr4* and *Tlr7* interacted with IRGs (Figures 6D–F).

### Cortex-specific transcriptional profiles were validated by qRT-PCR and immunohistochemistry

**FIGURE 7:**
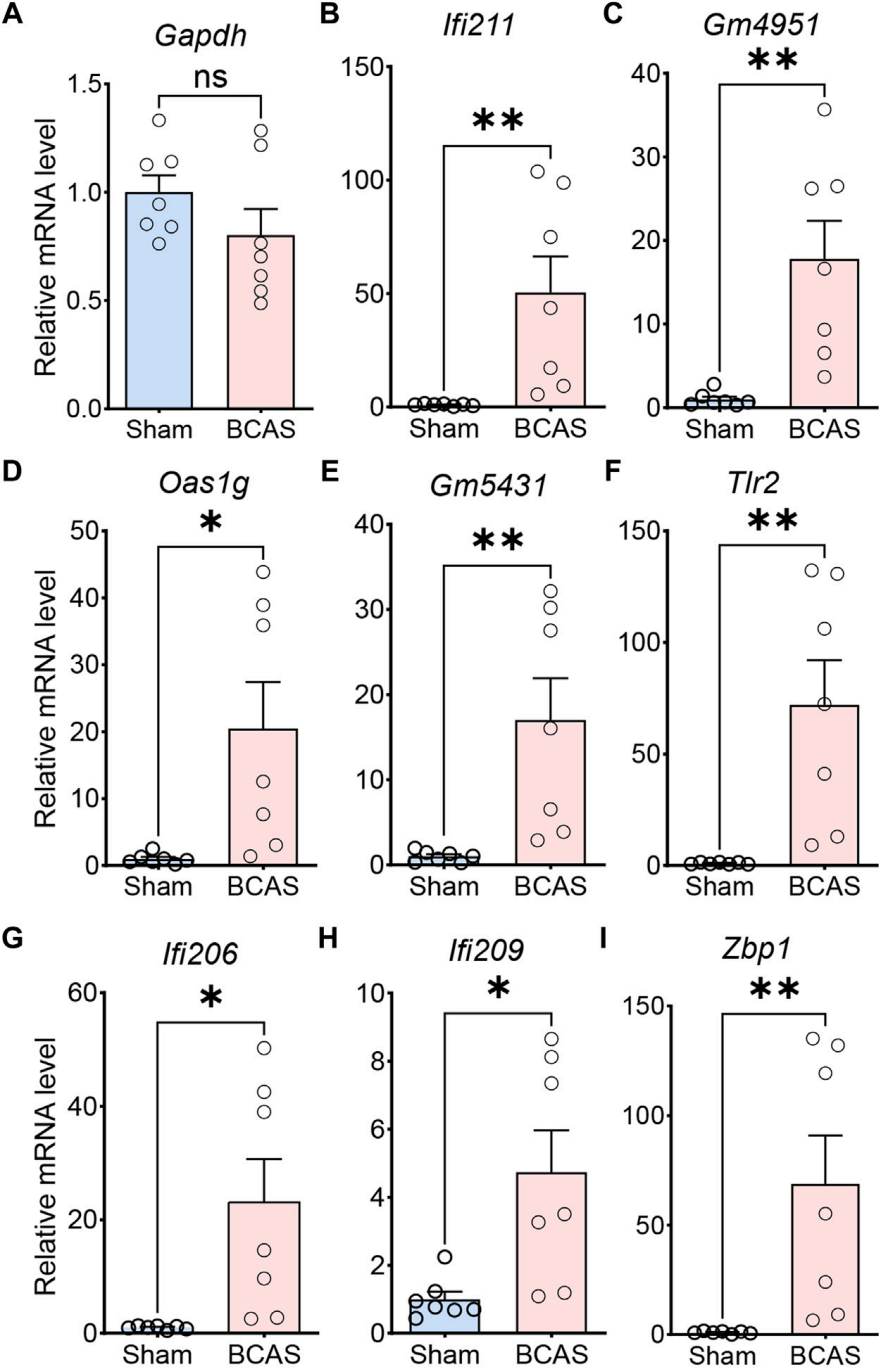
Validation of differential gene expression by quantitative RT-PCR. The relative mRNA expression levels of representative genes in cerebral cortex of sham and BCAS mice, as detected by qRT-PCR with GAPDH as the reference gene. **(A)** Note that the relative mRNA level of GADPH does not change between sham and BCAS mice. Up-regulated gene involved in “response to interferon-beta (*Ifi211*, *Gm4951*, *Oas1g*, *Gm5431*)” **(B–E)**, “activation of innate immune response (*Tlr2*, *Ifi206*, *Ifi209*, *Zbp1*)” **(F–I)**. Data are expressed as mean ± SEM, Unpaired t test (two-tailed), *p < 0.05. **p < 0.01 versus sham, n = 7 mice for each group.

We performed qRT-PCR to detect the expression of genes extremely enriched in the pathways of IFN-related neuroimmune signaling, including “response to interferon-beta” (*Ifi211*, *Gm4951*, Oas1g, Gm5431), and “activation of innate immune response” (*Tlr2*, *Ifi206*, *Ifi209*, *Zbp1*). Meanwhile, the expression of the housekeeping gene (*Gapdh*) was unchanged (Figure 7A). Compared with sham group, the BCAS-hypoperfusion mice induced enhanced mRNA levels of these genes in the cerebral cortex, which was consistent with our RNA-seq results (Figures 7B–I). Then, we tested expression levels of IFITM3 (IFN-inducible protein) at the protein level by immunohistochemistry in sham and BCAS mice. The results showed that IFN signaling pathways were also upregulated at the protein level following BCAS-hypoperfusion (Supplementary Material S10).

## Discussion

In clinical practice, there is a lot of causes for CCH, including cerebral small vessel disease, genetic factors, heart failure, cardiac arrhythmia and carotid artery stenosis. Long-term cerebral hypoperfusion could lead to vascular cognitive impairment, and even stroke. Currently, in preclinical experimental studies, many researchers have explored various animal models to mimic patients’ cerebral hypoperfusion status, including mice, rats and non-human primates’ animal models (Woodruff et al., 2011; Tukacs et al., 2020). These models include BCAS model, bilateral common carotid artery occlusion (BCCAO) model, left vertebral artery and bilateral internal carotid artery occlusion (three-vessel occlusion; 3VO) model and others. Nevertheless, none of these animal models fully recapitulate the human CCH phenotype, which clinically presents with cognitive deficits, motor dysfunctions as well as neuro-histopathological damages. In our current study, we developed a 0.16/0.18 mm BCAS mouse model, because it had some advantages. First of all, operators can manipulate the carotid artery stenosis degrees to control CBF by choosing different inner diameters of microcoils. For example, by using microcoils greater than or equal to 0.18 mm, BCAS operation fails to induce gray matter lesions in mice, whereas the application of a smaller inner diameter does, accompanied by significantly higher mortality rate. Secondly, there are different microcoils size selection strategies for mice’s bilateral artery, and the asymmetrical ischemia of right and left hemispheres induced by 0.16/0.18 mm BCAS procedure is closer to the clinical situation. Thus, in our study, we chose this kind of microcoil size for the establishment of our animal model with cerebral hypoperfusion. Thirdly, according to previous studies and our results, this mouse model showed good reproducibility in various phenotypes, including minor infarcts in the cortex (Figures 2A, B), microglial activation (Figures 3A,B). Besides, this model did not induce motor dysfunction (this was evidenced by the normal swimming speed of BCAS mice compared to the sham group), but resulted in spatial cognitive deficits in the Morris water maze test (Figures 1F, G; Supplementary Material S1). This helps us to further study cognitive function changes induced by BCAS-hypoperfusion, because it is often hard for researchers to distinguish between motor dysfunction and cognitive deficits in mice.

Cognitive impairments in animal models are normally evaluated by various methods, including Y mazes, radial arm maze as well as MWM, and they reflect different functions of cognition and memory (Mota et al., 2019). According to the previous studies, the 0.18 mm BCAS model could lead to mild cognitive dysfunction in the 8-arm radial maze test at 4 weeks after BCAS operation, as evidenced by deficits only in working memory task but not in the spatial reference memory task (Qin et al., 2017). However, after long-term hypoperfusion (5–6 months), 0.18 mm microcoil also induced working and reference memory, as evaluated by Barnes and radial arm maze tests (Nishio et al., 2010). And both 0.16 mm and 0.16/0.18 mm groups showed significant cognitive impairment in spatial reference study and memory functions at shorter duration after BCAS operation. For example, both 0.16 mm induced working memory deficits at only 2 weeks after BCAS operation (Toyama et al., 2018). In our study, the 0.16/0.18 mm BCAS mouse model displayed obvious deficits in spatial cognitive function in MWM test (Figures 1F, G; Supplementary Material S1), which was in line with previous reports (Miki et al., 2009; Zhou et al., 2022). Therefore, persistent decrease in CBF could compromise memory processes and affect cognitive functions in BCAS-hypoperfusion mice.

Moreover, the cognitive impairment was often accompanied with related brain regions damages, and thus we next explored the histological changes post BCAS surgery. According to previous studies, both 0.18 mm microcoils for 4 weeks only induced working memory because CCH at this condition only led to demyelination but not cortical or hippocampal lesions, while long-term CCH (5–6 months) could result in hippocampal atrophy (Nishio et al., 2010). Microinfarcts, minor infarcts and moderate infarcts were observed in BCAS group 3 weeks after hypoperfusion (Figure 2; Supplementary Materials S2, S3), which was consistent with our behavioral results. And previous researches also demonstrated that the infarct regions were randomly distributed in the whole brain post BCAS operation (Miki et al., 2009). Furthermore, our work revealed cortical lesions were usually accompanied with microglial activation and neuronal loss following BCAS-hypoperfusion. It is commonly known that glia activation (including microglia) can result in phenotypic and functional diversity, which may have beneficial or detrimental effects on the local neurons (Taylor et al., 2020). Specifically, appropriate activation of microglia can be beneficial to neuronal survival, but excessive activation of microglia may aggravate neuronal damage (Pietrucha-Dutczak et al., 2017; Su et al., 2019; Werner et al., 2020). CD68 is a lysosomal protein with high expression levels on macrophages and activated microglia, but low expression levels on resting microglia. Here, CD68/Iba1 double-staining was conducted and we revealed some of microglia/macrophages displayed phagocytosis phenotypes upon the status of highly activated after hypoperfusion injury. We found the cells positive for the general marker Iba-1 and the phagocytosis marker CD68 were clearly visible in the 0.16 mm side of BCAS mice, but not in the 0.18 mm side or sham mice (Supplementary Material S5). Then, we further observed the activation state to examine the beneficial or deleterious effects of microglia on neuronal survival. As reported, we found microglia could be significantly activated and engulf the local neurons at higher magnification after chronic hypoperfusion (Figure 4 **)**, characterized by hypertrophic morphology, with thickened and retracted processes (Qin et al., 2017). Therefore, the attenuation of microglia-mediated inflammatory cascades can be beneficial under such pathological conditions.

According to previous studies, microglial activation and the release of the inflammatory cytokine peak at the mid-late phase after ischemic insults, followed by a steady decline (Qin et al., 2017; Kim et al., 2020). And in our study, we found that significant brain damage and microglial activation had occurred, so we chose this time point for cortex-specific bulk RNA-seq profiling in order to capture the key genes and pathways associated with CCH. Considering the fact that DESeq2 was a R package with function of correction and normalization, and limma could be used instead. We presented results from these two analysis methods (Supplemntary Tables S2, S3). Indeed, the findings of both approaches appeared to be similar and equally persuasive. The work revealed the upregulated genes were extremely enriched in the IFN-related pathways following BCAS-hypoperfusion using GSEA analysis. In addition, GSEA analysis also identified significant upregulation of genes involved in the “activation of innate immune response” pathways. Interferon activated gene 209 (*Ifi209*), also known as PYHIN1, acting upstream of or within cellular response to interferon-beta, plays an important role in innate immunity (Andreoletti et al., 2015). Therefore, our findings are consistent with prior work that innate immune response is considered an essential step in the progression of chronic ischemia injury (Wu et al., 2017).

Our study was the first to identify the essential roles of IFN-mediated neuroimmune signaling post chronic hypoperfusion. IFN-signaling pathways, the critical components of the body immune system, can be divided into three subtypes, two of which perform opposite functions: type I (IFN-alpha and -beta) with pro-inflammatory activity and type II (IFN-γ) with anti-inflammatory activity. In our study of hypoperfusion-induced cortex-specific transcriptomes, remarkably, previously unreported IRGs were identified. While a significant role for type I IFN signaling is recognized in fighting central nervous system infections, type I IFN signaling has received limited study in chronic cerebral ischemic injury. We revealed that *Irf7* was extremely upregulated in this BCAS-hypoperfusion model and involved in stimulator of interferon genes (STING)-mediated IFN-beta signaling (Supplementary Table S1). STING functions by sensing cytoplasmic DNA and activates key transcription factors, including interferon regulatory factor 7 (IRF7), to initiate type I IFN expression (Banete et al., 2018; Chen et al., 2022). The STING signaling exert critical regulatory function in immune inflammatory response through the induction of cytokines, primarily type I IFNs. And the role of STING signaling in modulating neuroinflammation have been reported (Chin, 2019; Fryer et al., 2021; Paul et al., 2021; Li T et al., 2022). Moreover, IRF7 could greatly enhance STING-mediated IFN-beta promoter activity (Cheng et al., 2019; Dalskov et al., 2020). IRF7 is central to the production of type I IFN and activation of IRF7 is controlled by phosphorylation events, resulting in the formation of homodimers that are transcriptionally active. We speculated that the IRF7-related regulatory network contributed to the transcriptional activation of IRGs after cerebral hypoperfusion. It will be of great significance to investigate how IFN-mediated neuroimmune responses may participate in chronic cerebral ischemic injury mechanisms in future work.

While no studies have carefully explored the type I IFN responses in the BCAS-hypoperfusion mice so far, activation of IFN signaling has been determined in multiple rodent models of brain injury. Growing evidence has shown that type I IFNs can promote the release of pro-inflammatory factors and participate in regulating brain injury, which involve in interactions between different cell types in brain (Barrett et al., 2020; Todd et al., 2021). For example, mitochondria DNA (mtDNA) damage and release could play an important role in ischemic cascades. The damaged mitochondria could be exchanged from different cell types, and released into the cytoplasm of certain cell type, such as microglia (Todd et al., 2021). Moreover, according to the previous studies, STING was able to complex with the damaged mtDNA, and activated the downstream cyclic GMP-AMP synthase (cGAS)-STING immune pathways (Davis et al., 2014; Hayakawa et al., 2016). Further, the activation of the cGAS-STING pathway could promote the formation of a pro-inflammatory microenvironment and the cGAS-STING expression in microglia was upregulated after stroke (Kong et al., 2022), and conditional knockout of cGAS in microglia attenuates ischemic/reperfusion-induced brain injury (Liao et al., 2020). Similarly, cGAS knockdown could promote microglial M2 polarization to alleviate neuroinflammation by inhibiting cGAS-STING pathway both in an oxygen-glucose deprivation cell model and a middle cerebral artery occlusion (MCAO) mouse model (Jiang et al., 2021). In addition, in the traumatic brain injury (TBI) rodent model, excess IFN-beta was released from the “sick neuron” under the endoplasmic reticulum stress conditions *via* STING signaling, and enforced activation and pro-inflammatory M1 polarization of microglia, which resulted in secondary brain injury by recruiting more infiltration of peripheral immune cells (Sen et al., 2020). Interestingly, the shared transcriptional changes of microglia and astrocyte of TBI were enriched in type I IFN signaling (Todd et al., 2021). This suggested that the neuroinflammation response involved in IFN signaling of TBI model is not limited to microglia, but also to astrocyte, another kind of important cell population responsible for brain insults. More importantly, a recent study demonstrated that global IFN-beta deficiency [IFN-beta (−/−)] led to reduced neuroinflammation and improved neurological functions in TBI mice (Barrett et al., 2020). Also, our results suggested that IFN signaling pathway was upregulated both at the RNA level and the protein level post BCAS-hypoperfusion (Figure 7; Supplementary Material S10). In some study about neurodegeneration, such as Alzheimer’s disease, investigators have found that IFITM3 can act as γ-secretase modulatory protein and increase the deposition of amyloid-beta in brain *via* inflammatory mechanisms (Hur et al., 2020). Besides, the level of IFITM3 was elevated under schizophrenia and other inflammatory conditions in the brain (Horváth and Mirnics, 2014). And studies have identified IFITM3 as a new player involved in microglial phenotype modulation in response to a variety of inflammatory conditions (Harmon et al., 2022). Therefore, understanding changes in the type I IFN signals would help to identify potential cellular and molecular mechanisms for hypoperfusion-induced brain injury.

TLRs that are highly expressed in microglia, are known to play a prominent role in the sustained ischemic stroke. Another salient finding in the current study was that the classic TLR signaling appeared to interact with IFN-signaling after hypoperfusion. In fact, TLR9 inside endolysosome can act as the sensor of double-strand DNA (dsDNA) or self-DNA released from dying cells, and contains an important leucine-rich repeat domain responsible for cytosine-phosphate-guanosine (CpG)-DNA binding, receptor oligomerization, and sequential signaling transduction. Then, after MyD88 adaptor protein is recruited by a Toll/IL-1 receptor domain of dimerized TLR9, the LC3-TLR9 interaction provides with an anchor to induce type I IFN expression *via* the activation of IRF7 (Julian et al., 2012; Briard et al., 2020). Likewise, intracellular nucleic-acid can also induce the production of IFN-beta *via* nuclei-acid sensors signaling pathways, including TLR7/TLR8-MyD88 signaling pathway, TLR3-IFN-beta signaling pathway and so on in autoimmune diseases (Gilliet et al., 2008). Interestingly, accumulating evidence has shown that systemic administration of CpG oligodeoxynucleotides can activate TLR9 (Stenzel-Poore et al., 2007; Stevens et al., 2008; Marsh et al., 2009b; Bahjat et al., 2011). And, CpG preconditioning can reprogram the response of the brain to the subsequent ischemic stroke challenge dominated by the high production of Type I IFN, and as a result, play protective role by minimizing ischemic damage. It seems that the protective role of IFN mentioned here is inconsistent with the harmful role mentioned in the last paragraph (Barrett et al., 2020), yet this could be partially explained by the difference between knockdown/knockout and preconditioning treatments of IFN. Thus, we speculated that like the classic MCAO mouse model, the crosstalk of TLR signaling and IFN signaling played an important role in the progression of CCH.

As a matter of fact, in the 1960s, Dahl and Balfour found that a pre-exposure to a short-term anoxia challenge to the rat brain could lead to an increase in anaerobic glycolysis, and as a result, the survival rate could improve after a subsequent anoxic exposure (Dahl and Balfour, 1964). Later, neurobiologists refer to this experimental phenomenon as “ischemic preconditioning” (IPC), which means that prior exposure to non-fatal and subthreshold cerebral preconditioning stimulus can transiently increase ischemic tolerance to sequentially cerebral ischemic injury (Gidday, 2006). Various signaling pathways have been found to be involved in the protective role of IPC, including mitochondrial function, metabolism, hypoxia-inducible factor (HIF-1) signaling pathways, vascular endothelial growth factor (VEGF) signaling pathways and so on (Li et al., 2017). Interestingly, recent studies have also shown TLR signaling pathways and IFN-regulated genes pathways represent an attractive therapeutic target involved in IPC for ischemic stroke (Marsh et al., 2009a; Stevens et al., 2011; McDonough et al., 2020). For example, one study revealed that the investigator found pre-conditioned with Gardiquimod (a TLR7 agonist) could significantly reduce infarct volume in MCAO model, but this protective effect was abolished in IFNAR(−/−) MCAO model, suggesting that IPC induced by TLR7 resulted in neuroprotective effect against ischemic insults *via* IFN-mediated signaling pathway (Leung et al., 2012). Because both IFN and TLRs signaling pathways were upregulated in our animal model and they existed some crosstalk at the molecular levels (Figures 6C–F), treatment with pharmacological agents in combination with these two pathways seems like a more promising approach of IPC. Excitingly, approved by the FDA, IFNβ has been used for the treatment of multiple sclerosis (MS) in clinical medicine as a cytokine with immunomodulatory properties (Cheriyan et al., 2012; Kasper and Reder, 2014). Also previous studies have indicated that IFNβ treatment ameliorated ischemic stroke in MCAO model (Kuo et al., 2020), suggesting that it had great value for clinical translational applications for chronic cerebral hypoperfusion. In addition, on the one hand, published astrocyte-specific single-nuclei RNA sequencing (snRNA-seq) data also showed the IFN-signaling pathways increased in non-human primate preclinical primate model, suggesting that the role of IFN signaling pathways in ischemic stroke appears to be evolutionarily conserved mechanism. On the other hand, D192935 (a TLR9 agonist) preconditioning could efficiently reduce ischemic insults in a non-primate preclinical animal model (Stevens et al., 2019). More importantly, although the concept of IPC was firstly raised according to preclinical animal models, yet retrospective studies have shown that transient ischemic attacks induced IPC-like protective effects on patients (Moncayo et al., 2000; Wegener et al., 2004). However, it is neither ethical nor feasible to apply some direct transient ischemic challenges on the brains of those patients with high stroke risk factors, because it is difficult to distinguish between “harmful” and “beneficial” IPC-like challenge. Instead, it is a more promising strategy to investigate underlying mechanisms of IPC and design some drug targets mimic IPC. Indeed, CCH can also be seen as a kind of IPC, so the upregulated pathways identified in our RNA-seq can not only represent drug targets for CCH, but also become some potential biopharmaceutical targets to mimic IPC against other more severe brain insults, such as stroke, TBI and so on. In details, these inspiring drug targets of IPC include type I IFNs (such as IFI209), TLR-mediated intracellular downstream molecules (MyD88) as well as non-TLR pattern recognition receptors (STING). Encouragingly, it will be more easily to achieve translational and clinical research when modulating some specific molecular targets by using some novel molecular approaches or novel nanoparticle technologies (Wieghofer et al., 2015; Nance et al., 2017).

Our study provides a novel dataset of gene expression profiles in mouse cerebral cortex after BCAS-hypoperfusion. However, there are some important limitations. First, it is expected that significant changes of transcriptomic profiles in some cell types may have been missed. Therefore, it will be interesting to investigate cell-specific transcriptomic profiles in future work. Second, future studies will be needed to clarify whether IFITM3 has the capacity to modulate microglial phenotype in conditions of chronic ischemic brain injury. Also, our transcriptomic data lacked further validation on functional levels. Given these limitations, future studies should be well designed, taking functional verification into consideration. At last, our current study is somewhat preliminary and descriptive. In the light of the role of IFN signaling in cerebral ischemia remains controversial, there is still a long way to go before basic research results can be translated into clinical results.

In summary, this preliminary study is the first to provide valid evidence for the cortex-specific gene-expression profiles following chronic cerebral ischemic injury. Our findings suggest that increased levels of gene expression related to IFN-mediated neuroimmune signaling pathway may exert a critical impact on the occurrence and development of cerebral hypoperfusion. Our RNA-seq data provide a valuable resource for future investigations into the roles of IRGs in hypoperfusion-induced neuropathological changes.

## Data Availability

The datasets presented in this study can be found in online repositories. The names of the repository/repositories and accession number(s) can be found below: https://www.ncbi.nlm.nih.gov/, GSE210666.
