# Supplementary material for: Cortex-specific transcriptome profiling reveals upregulation of interferon-regulated genes after deeper cerebral hypoperfusion in mice
Source: Front Physiol. 2023 Mar 13;14:1056354. doi: 10.3389/fphys.2023.1056354 (PMC10040763; doi:10.3389/fphys.2023.1056354)
Supplement: Supplementary file 4 [file Image1.pdf]

Supplementary Material

Supplementary material 1. Behavioral analyses of sham and BCAS mice after the operation. (A) swimming speed and (B) Escape latency of sham and BCAS mice in MWM test (learning phase). MWM, Morris water maze test. Unpaired t test (two-tailed), *p<0.05, **p<0.01, n = 8-10 mice per group.

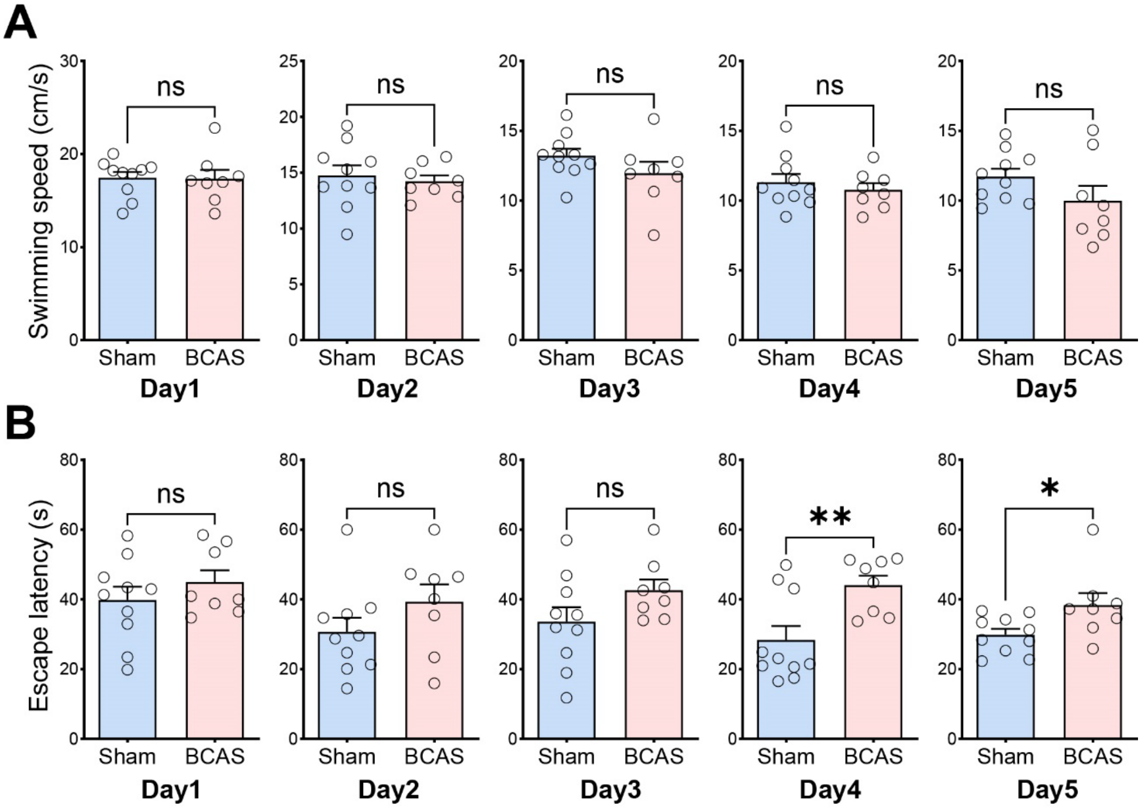

Supplementary material 2. Brain damage at different sections induced by cerebral hypoperfusion 3 weeks after the operation. Representative images of Hematoxylin-stained coronal sections depicting the morphology of corpus callosum (A), striatum (A, B), cortex (A-D) and hippocampus (D).

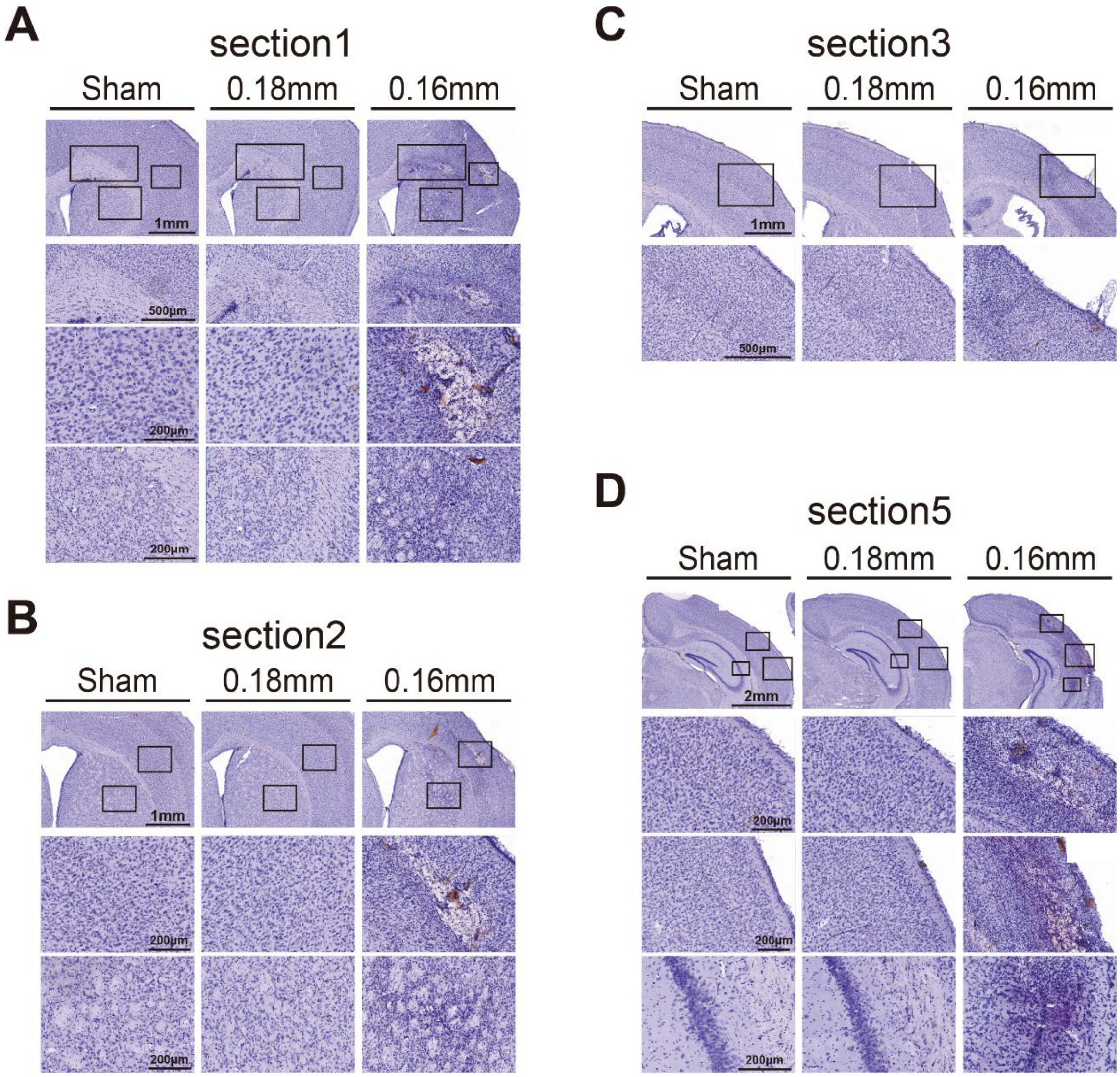

Supplementary material 3. Histological changes induced by cerebral hypoperfusion 4 weeks after BCAS operation. (A) Hematoxylin-stained consecutive coronal slices to detect brain injury after 4 weeks cerebral hypoperfusion. (B) Bar plots for percentage of infarct size of each individual section. Each point represents the mean value of per section of 8 BCAS mice. Data are expressed as mean±SEM. Paired t test (two-tailed), *$p<0.05$, versus 0.18mm side, n=8-10 mice per group.

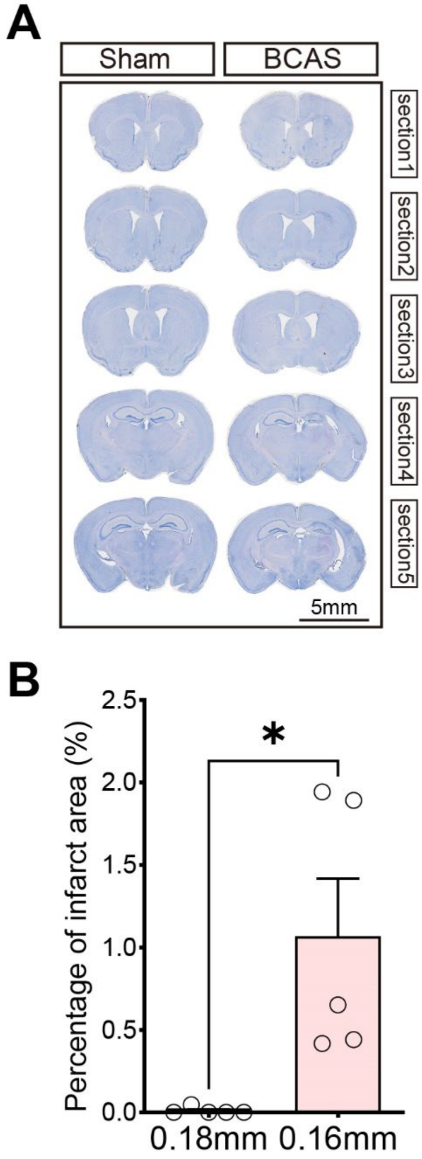

Supplementary material 4. Activation of microglia due to cerebral hypoperfusion 3 weeks postoperation. Representative pictures of Iba-1 labeled sections revealing the microglial activation in the corpus callosum (A), striatum (A-C), cortex (B-D) and hippocampus (D).

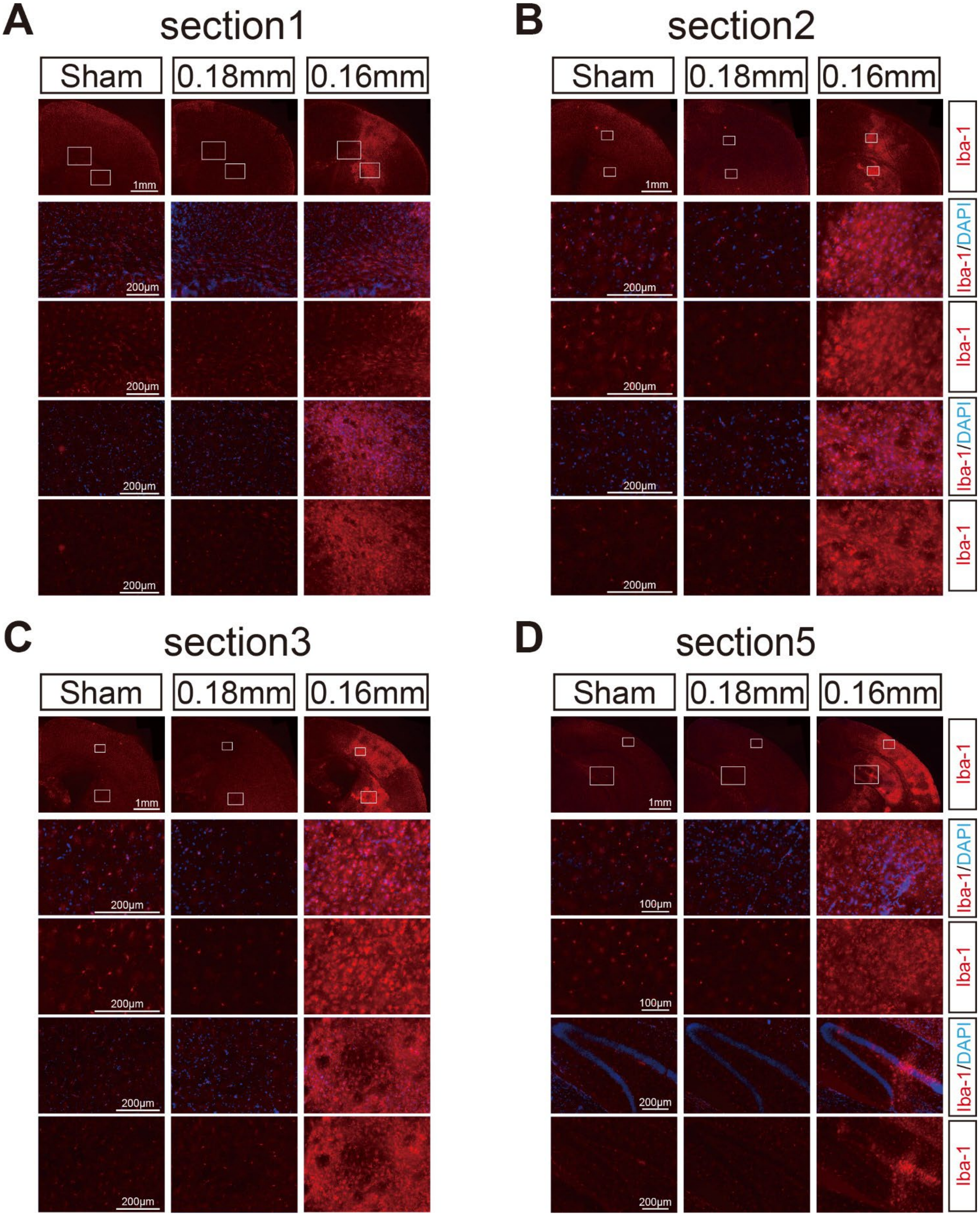

Supplementary material 5. Microglial phagocytic activity was examined by Iba1/CD68 double-staining after BCAS-hypoperfusion**. (A)** Representative confocal images labeled with Iba-1/CD68. NC, negative control without the CD68 primary antibody. Background staining is that of the secondary antibody alone. (**B**) Representative images at higher resolution of Iba1/CD68 immunofluorescence showing microglial cells positive for CD68 (arrowheads) in BCAS mice. The data rule out the background caused by the secondary antibody, as evidenced by NC.

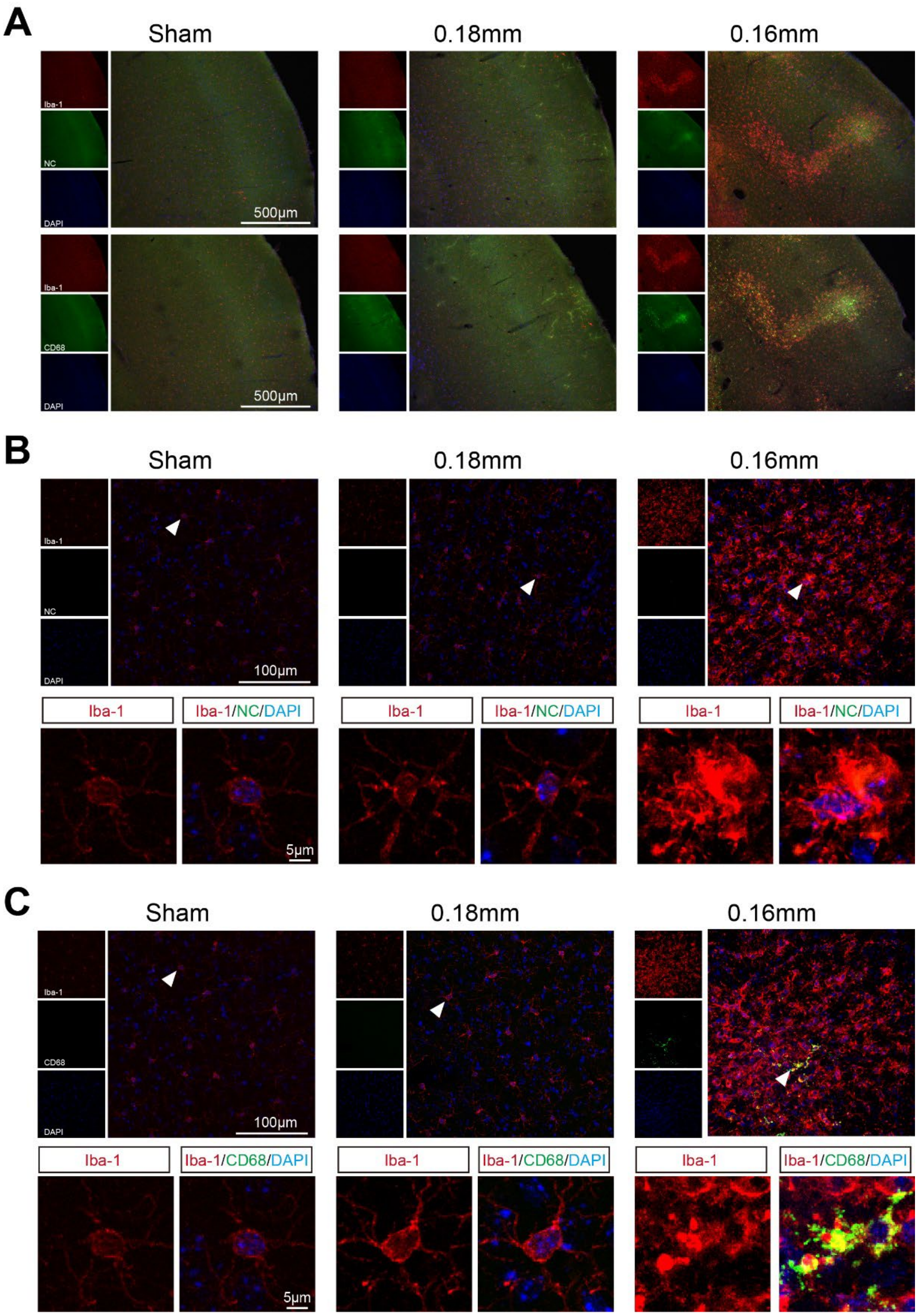

Supplementary material 6. Cortex-specific pathway analysis of transcriptome changes by limma after BCAS-hypoperfusion. (A-D) (Up) Enrichment plots and (Bottom) IGV map tracks for representative genes in the up-regulated and down-regulated pathways. Notice increases of RNA-seq signal for "positive regulation of innate immune response" genes (*Itgp*, *Il12b*) and "positive regulation of response to biotic stimulus" genes (*Hmgb2*, *Fadd*) in BCAS compared to sham, accompanied by decreases of RNA-seq signal for neuronal-activity related genes (*Rgs4, Stx1a, Nrn1, Chrnb3*). NES, Normalized Enrichment Score.

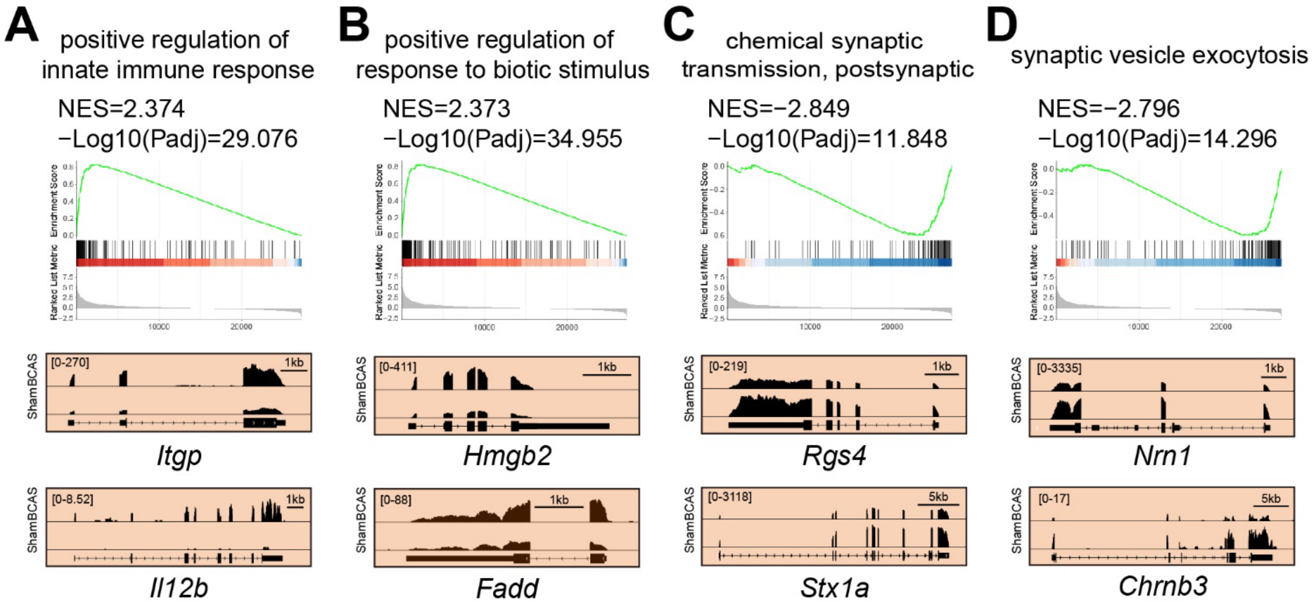

Supplementary material 7. Cortex-specific pathway analysis of transcriptome changes by DESeq2 after BCAS-hypoperfusion. (A) PCA plot of cortex-specific RNA-seq in sham and BCAS mice. N=4 for each group. (B) Up-regulated and down-regulated functional pathways analyzed using GSEA (BCAS vs. Sham). Notice high enrichment for IFN-beta signaling pathways. (C-F) (Up) Enrichment plots and (Bottom) IGV map tracks for representative genes in the up-regulated and down-regulated pathways. Notice increases of RNA-seq signal for IFN-stimulated genes (*Irgm1, Ifi204*) and antigen processing and presentation genes (*H2-Aa, Trem2*) in BCAS compared to sham, accompanied by decreases of RNA-seq signal for neuronal-activity related genes (*Nptx2, Grin1, Stxbp1, Syt5*). NES, Normalized Enrichment Score.

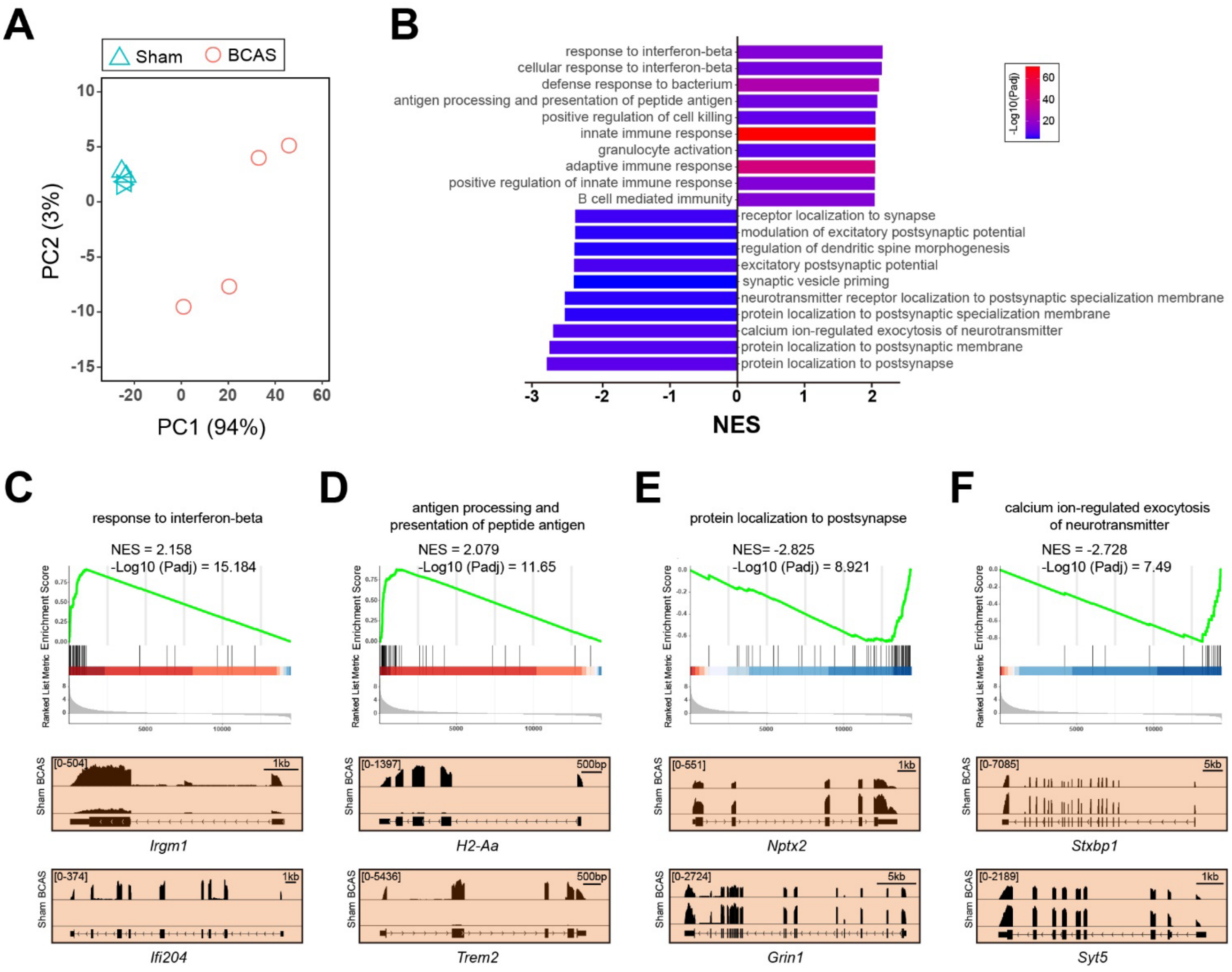

Supplementary material 8. Cortex-specific IPA analysis of DEGs by DESeq2 post cerebral hypoperfusion. (A) Volcano plots indicated genes with differential expression as defined by an adjusted P value < 0.05 and a |Log2FC| > 1 in the cerebral cortex after BCAS procedure (BCAS vs. sham). Points highlighted in red: significant differentially up-regulated expressed gene; Points highlighted in blue: significant differentially down-regulated expressed gene; Gray dot: non-significant differentially expressed gene. (B) Bar plot showing the top canonical pathways significantly enriched in the BCAS-hypoperfusion mice. Orange bars: positive regulatory pathway; Gray bars: no activity pattern available. (C-D) Upstream regulatory analysis of DEGs revealed that IRF7-dominated hub genes are pointed to be the top upstream regulators in IPA effect networks. (C-E) TLR4, TLR7 and STING1 also participated in and coordinately regulated IRF7-mediated IFN signaling. STING1, the gene that encodes STING. (F) IGV map tracks for representative genes in the top canonical pathways. Notice increases of RNA-seq signal for TLRs-related IRF7 and IFN-regulated genes (*Irf7, Sting1, Tlr4* and *Tlr7*) in BCAS compared to sham.

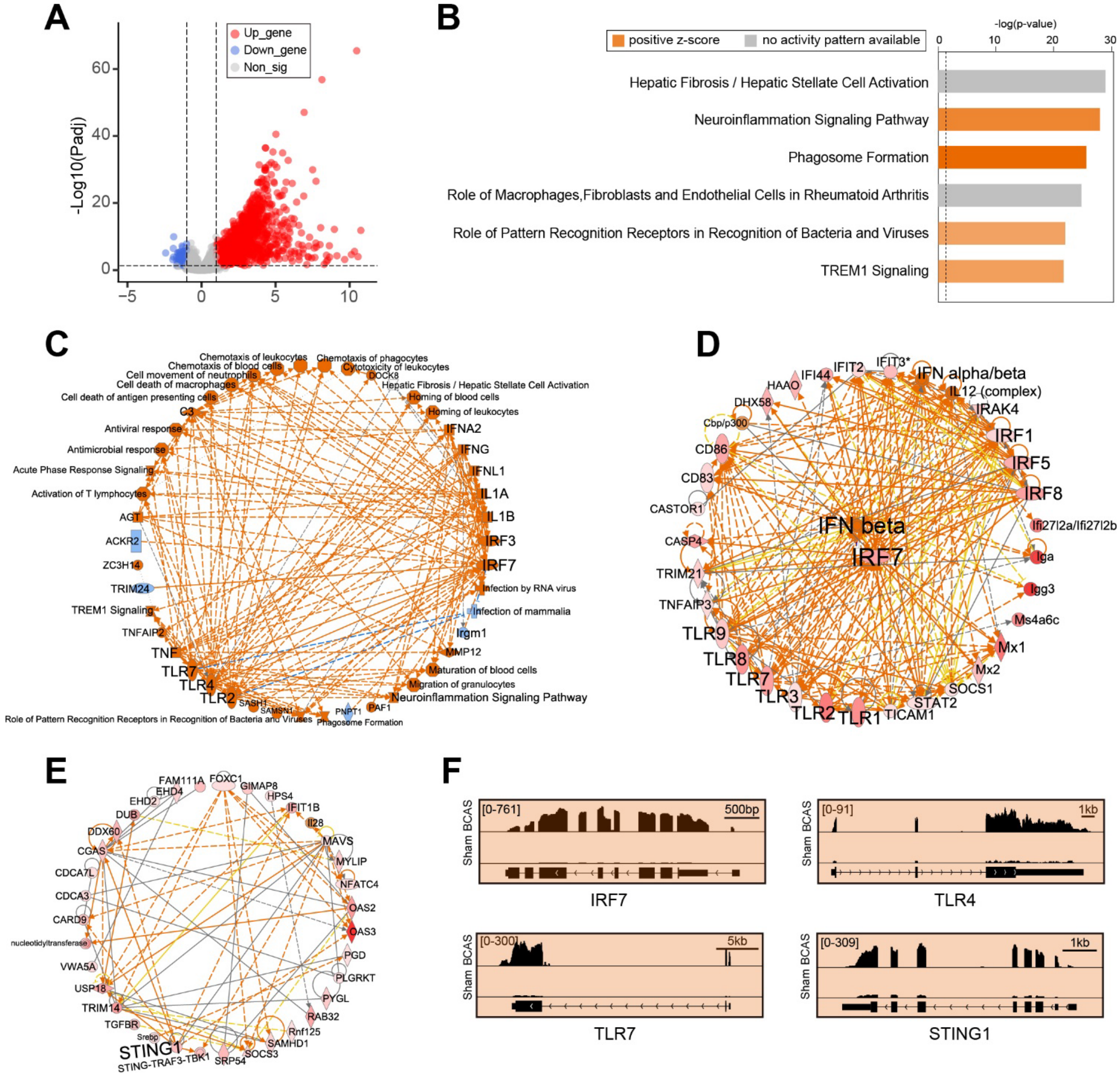

Supplementary material 9. Validation of DESeq2 results by quantitative RT-PCR analysis after chronic hypoperfusion. The relative mRNA expression levels of representative genes in cerebral cortex from BCAS and sham groups, as detected by qRT-PCR with GAPDH as the reference gene. Note that the relative mRNA level of GADPH does not change between sham and BCAS mice. Up-regulated genes involved in "antigen processing and presentation of peptide antigen (*Trem2, Fcgr2b, H2-Aa, H2-Ab1, Ctsl*)" (A), "Response to IFN-beta (*Irgm1, Irgm2, Gbp2, Ifnar2, Irf1, Ifi204*)" (B), and "Potential upstream regulators and canonical pathways obtained from IPA analysis (*Irf7, Sting1, Tlr4, Tlr7*)" (C). Data are expressed as mean±SEM, Unpaired t test (two-tailed), *p<0.05, **p<0.01 versus sham, n=5 mice for each group.

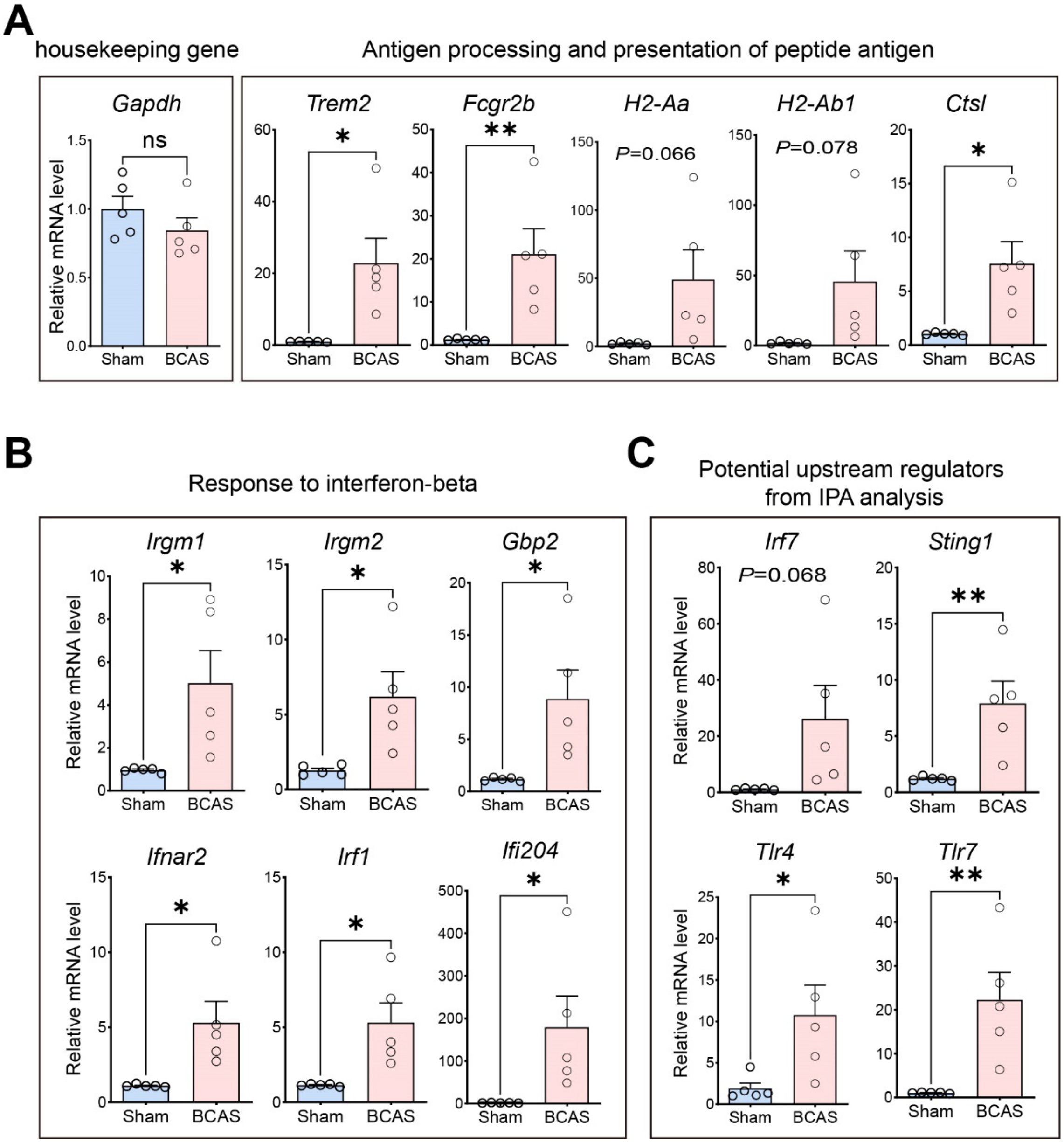

Supplementary material 10. Validation of the differential expression of IFITM3 at protein level in the two groups by immunohistochemistry. Representative images of IHC of brain sections labeled with IFITM3 in sham and BCAS mice. NC, negative control without primary antibody. IFITM3, IFN-inducible protein.

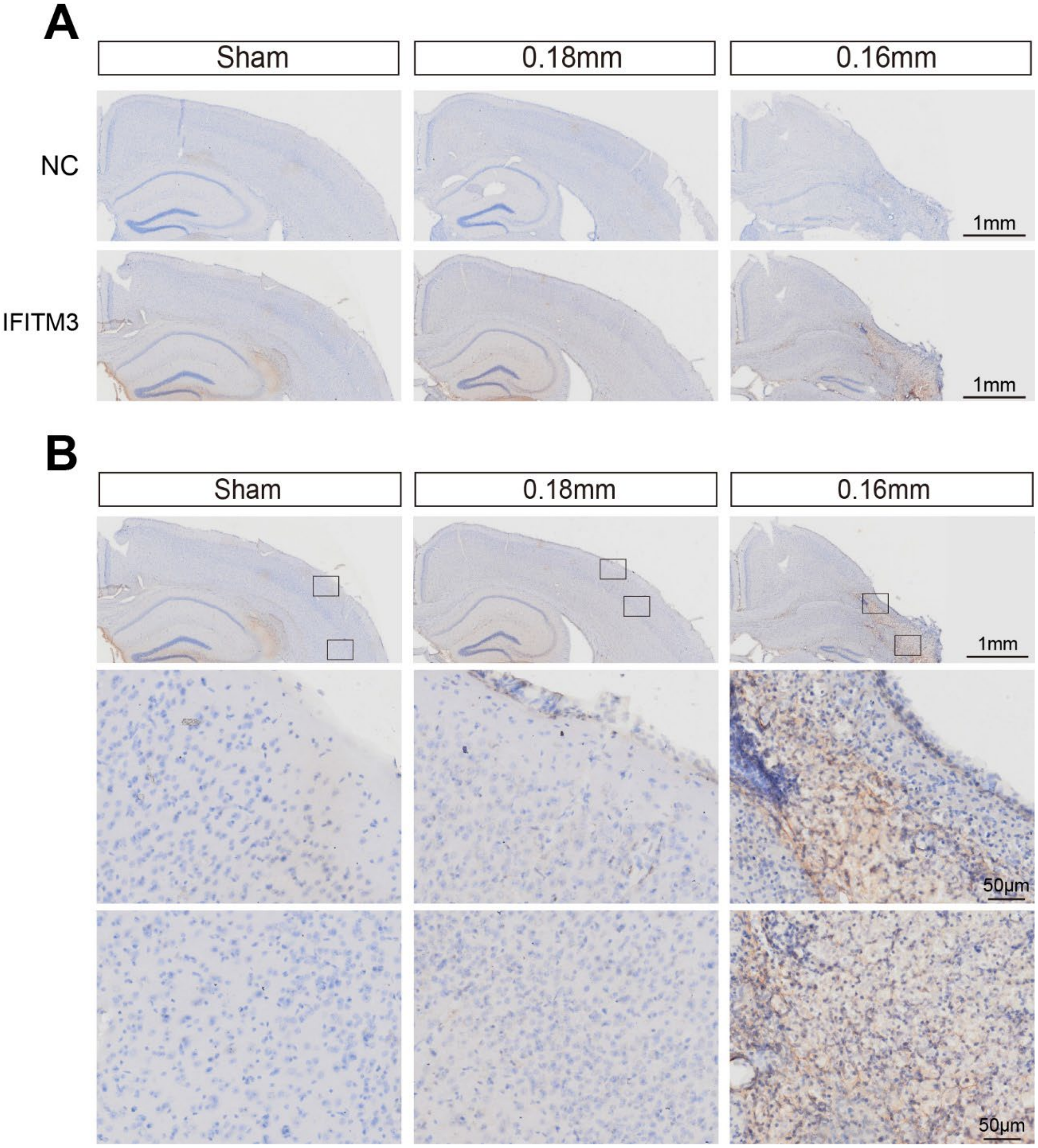

Supplementary material 11. Representative images for FastQC data quality control. (A) Basic statistics. (B) Per base sequence quality. (C) Per sequence quality scores. (D) Per sequence GC content. (E) Per base N content. (F) Adapter content.

A

Measure	Value
Filename	BCAS_3_1.fq.gz
File type	Conventional base calls
Encoding	Sanger / Illumina 1.9
Total Sequences	21371327
Sequences flagged as poor quality	0
Sequence length	100-150
%GC	50

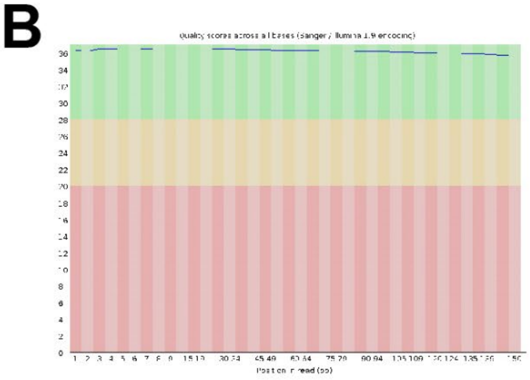

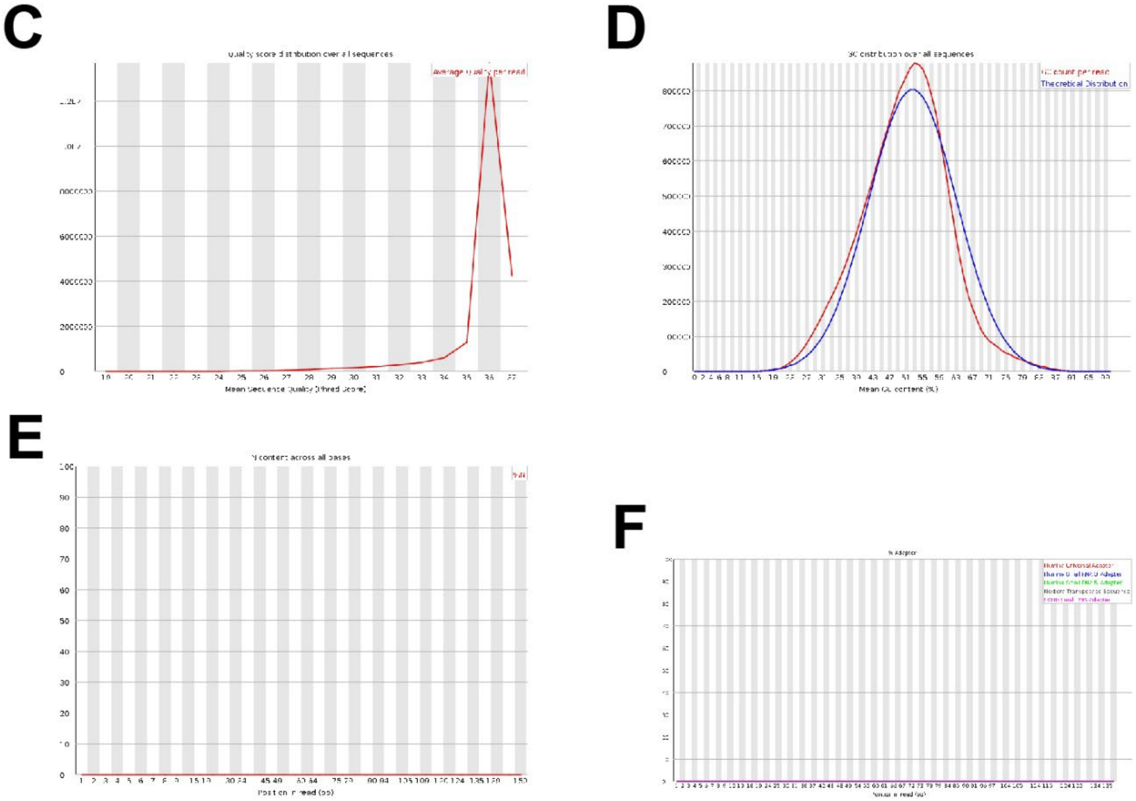

Supplementary material 12. List of primer sequences used for qRT-PCR analysis in validation of limma and DESeq2 results.

**Gene**	**Forward Primer**	**Reserve Primer**
Gapdh	AATGTGTCCGTCGTGGATCTGA	GATGCCTGCTTCACCACCTTCT
Ifi211	CCAGTCACCAATACTCCACAGC	CTCTGAGTGGAGAACAGCACCT
Gm4951	GGACAAGCTGATAAGTGACCTCC	CAGCCAAGTCTTCCACCTCAGA
Oas1g	GAGTCTCATCCGCCTGGTCAAA	CCAGGCATAGACAGTGAGTAGC
Gm5431	CATTGACACGGTTGCAAGCGATG	AAAGGAGAGGGTCTGCTAGAGG
Tlr2	ACAGCAAGGTCTTCCTGGTTCC	GCTCCCTTACAGGCTGAGTTCT
Ifi206	GCGAAGATATTCCAGAACTTGC	CTGGAGGTAAGTGGAGGAGG
Ifi209	CTCCAGTGTCTTCAGGAACAGC	CCTTGCTGGTGACCATTTTCCTC
Zbp1	GATCTACCACTCACGTCAGGAAG	GGCAATGGAGATGTGGCTGTTG
Irgm1	CCTTCCCCTTTGTCACGTCATAC	CTCCTTGATTCCGTAGACCAGTT
Irgm2	ATCTCTCCACCTGGTTGCTCTG	TTCCAGTGCGATACAACCGAGC
Gbp2	ACAAGCTAGCTGGGAAGAGG	TTCAAGGCCCTCAGTGTCAA
Ifnar2	GAGCCTAGAGACTATCACACCG	TACCAGAGGGTGTAGTTAGCGG
Irf1	AACCAAATCCCAGGGCTGAT	GGAACAGACAGGCATCCTTG
Ifi204	GCCAGCCCTAAGATCTGTGA	TTTTCCACTCCCCACCACTT
Trem2	CTACCAGTGTCAGAGTCTCCGA	CCTCGAAACTCGATGACTCCTC
Fcgr2b	CTACTGTGGACAGCCGTGCTAA	TCACCGTGTCTTCCTTGAGCAC
H2-Aa	AAATTCCACCCCAGCTACCA	TCCACAAAGCAGATGAGGGT
H2-Ab1	TTTGCTTTCTGAAGGGGGCA	TCGCCCATGAACTGGTACAC
Ctsl	GTGGAGGAGAGCGATATGGG	TTCACCACCTGCCTGAATTC
Sting1	ATGTCACAGGATGCCAAAGC	ACAGTGAGAAACTGTTTCCGTC
Tlr4	TTCTTCTCCTGCCTGACACCA	TAAGCCATGCCATGCCTTGT
Irf7	AAGCATTTCGGTCGTAGGGA	TCTTCGCTCTCTTCGCTCAG
Tlr7	GAAGCTTTAGTGGACTCTCTGAC	CTTCGTGATGGAGAAGATGTTGT
